# High-Resolution Profiling of Stationary-Phase Survival Reveals Yeast Longevity Factors and Their Genetic Interactions

**DOI:** 10.1371/journal.pgen.1004168

**Published:** 2014-02-27

**Authors:** Erika Garay, Sergio E. Campos, Jorge González de la Cruz, Ana P. Gaspar, Adrian Jinich, Alexander DeLuna

**Affiliations:** Laboratorio Nacional de Genómica para la Biodiversidad, Centro de Investigación y de Estudios Avanzados del IPN, Irapuato, Guanajuato, Mexico; Stanford University Medical Center, United States of America

## Abstract

Lifespan is influenced by a large number of conserved proteins and gene-regulatory pathways. Here, we introduce a strategy for systematically finding such longevity factors in *Saccharomyces cerevisiae* and scoring the genetic interactions (epistasis) among these factors. Specifically, we developed an automated competition-based assay for chronological lifespan, defined as stationary-phase survival of yeast populations, and used it to phenotype over 5,600 single- or double-gene knockouts at unprecedented quantitative resolution. We found that 14% of the viable yeast mutant strains were affected in their stationary-phase survival; the extent of true-positive chronological lifespan factors was estimated by accounting for the effects of culture aeration and adaptive regrowth. We show that lifespan extension by dietary restriction depends on the Swr1 histone-exchange complex and that a functional link between autophagy and the lipid-homeostasis factor Arv1 has an impact on cellular lifespan. Importantly, we describe the first genetic interaction network based on aging phenotypes, which successfully recapitulated the core-autophagy machinery and confirmed a role of the human tumor suppressor *PTEN* homologue in yeast lifespan and phosphatidylinositol phosphate metabolism. Our quantitative analysis of longevity factors and their genetic interactions provides insights into the gene-network interactions of aging cells.

## Author Summary

The budding yeast *Saccharomyces cerevisiae* has emerged as an important model for the genetic analysis of aging, and insights gained about this process in yeast cells enhance our understanding of aging in other organisms, including humans. Even in yeast, our knowledge of the number and identity of the genes that determine lifespan is limited and we are still lacking a general picture of how different genetic aging factors work together. Here, using an innovative sensitive technique, we have characterized the stationary-phase survival of yeast single- and double-gene knockout mutants to screen for genes that control chronological aging and to score the genetic interactions among these genes. Our results showed that an important fraction (14%) of the genome contributes to the regulation of lifespan, including genes and pathways that had not been previously associated to this phenotype. We also constructed a genetic interaction map which recapitulated lifespan-determining pathways and highlighted genetic associations between the autophagy machinery and the phosphatidylinositol-phosphate and lipid-homeostasis pathways. Our study provides not only an accurate catalogue of yeast aging genes, but also a picture of the gene-network wiring of aging cells.

## Introduction

Lifespan is a complex trait with clear genetic underpinnings [1]. Mutations in the insulin/IGF-1 receptor gene *daf-2* increase the lifespan of *Caenorhabditis elegans* [2] and *Drosophila melanogaster* [3], while mice lacking the insulin receptor in adipose tissue live longer as well [4]. Studies on the budding yeast *Saccharomyces cerevisiae* have also been pivotal to discover conserved longevity mechanisms [5]–[7]. Aging of dividing cells has been modeled in yeast by its replicative lifespan, defined as the number of daughter cells produced before death [8]. In addition, chronological lifespan (CLS), the duration of yeast survival in stationary phase, provides a model of aging of non-dividing quiescent cells [9], [10]. Evidence to date indicates that both forms of yeast aging are modulated by the Tor/Sch9 [11], [12] and Ras/cAMP/PKA [13] pathways. Likewise, the downstream activity of proteins with roles in autophagy [14], redox balance [15], [16], and mitochondrial function [17], [18] influence lifespan from yeast to mammals. Moreover, dietary restriction delays both replicative and chronological aging in yeast; this non-genetic intervention has been linked to the down-regulation of Tor and Sch9 in response to nutrients [12], [19].

Current phenotyping strategies allow the characterization of yeast CLS in a high-throughput manner. Survival as a function of time in stationary phase has been estimated by monitoring the outgrowth of either individual-strain cultures in parallel [19], [20] or large pools of gene-deletion strains coupled to microarray [21], [22] or deep-sequencing analysis [23]. Such large-scale approaches have recently opened the door to the first genome-wide screens for lifespan factors [19], [21]–[23]. With these catalogs in hand, the next great challenge is to describe how different genetic factors are integrated to determine lifespan in yeast.

Quantitative genetic interaction (GI) analysis has great potential to shed light on the genetic architecture of the lifespan phenotype and the mechanisms that underlie the aging process. By depicting the ways in which individual mutations affect each other's phenotypic consequences, GIs inform whether genes act in common or independent pathways [24]. A number of breakthrough studies based on the large-scale GI analysis of cell-proliferation phenotypes (mainly colony growth) have provided important insights into the genetic organization of the living cell [25]–[31]. Multi-conditional and multi-phenotype GI maps have further helped to elucidate cellular pathways and responses to stimuli [32], [33] and, notably, GI networks retain conserved features and overall levels of pathway crosstalk across large evolutionary distances [34]. Yet, systematic double-mutant analyses of lifespan phenotypes and their ensuing GI networks are still missing.

In this study, we sought to investigate in a quantitative manner the genes and gene-network wiring that underlies yeast CLS. By using a novel profiling tool based on the competitive survival of yeast populations on stationary phase, we generated a high-resolution CLS catalogue of 3,878 single-knockout mutations and constructed the first systematic lifespan GI map derived from over 1,800 double-knockout phenotypes. In doing so, we revealed novel genetic aging factors and shed light on functional associations within and between lifespan-determining pathways.

## Results

### A sensitive high-throughput strategy to profile yeast chronological lifespan

**Figure 1:**
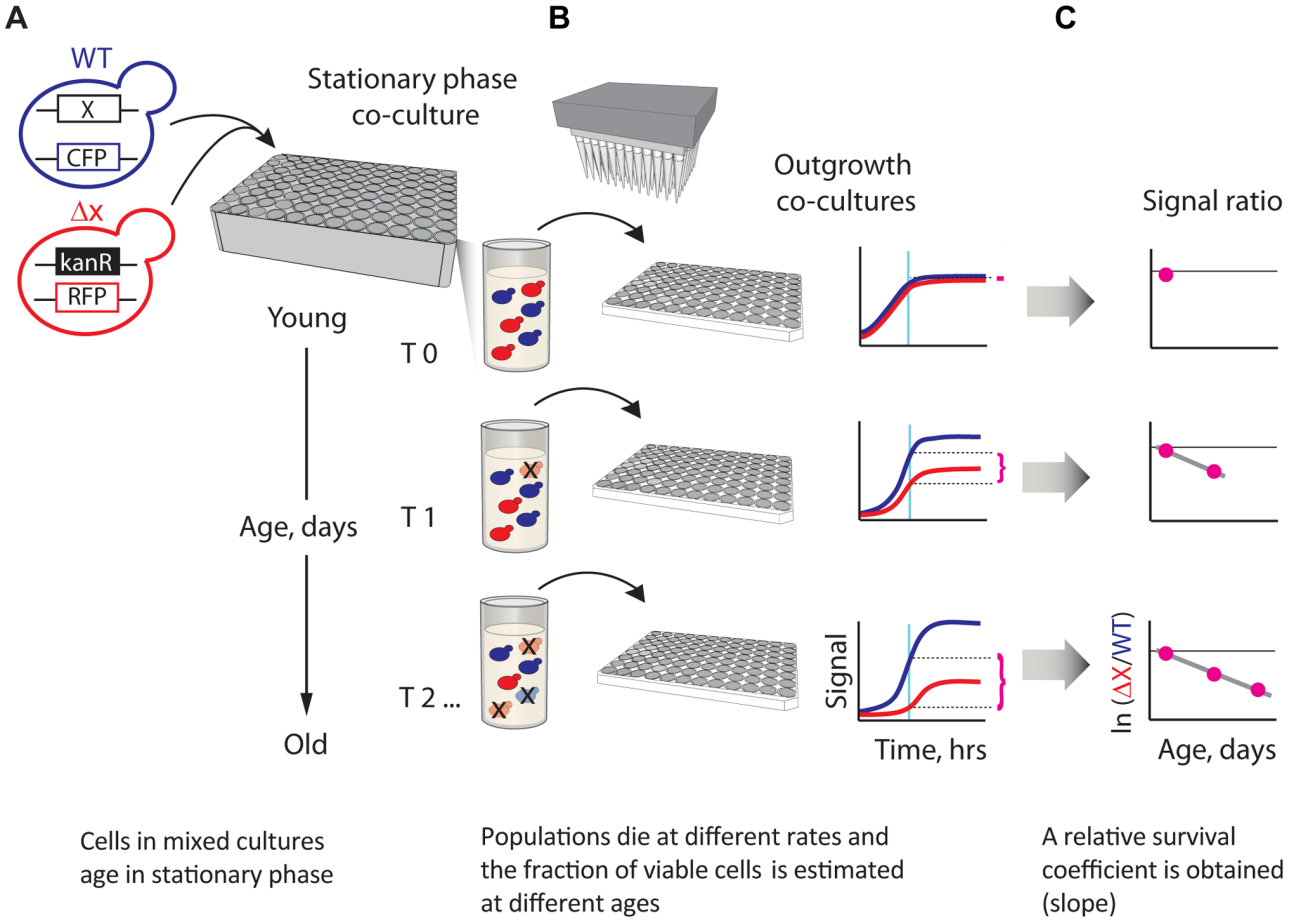
A novel strategy enabling quantitative genetic analysis of yeast CLS. (**A**) Schematic showing an RFP-labeled mutant, *x*Δ, that lives shorter than the CFP-labeled WT reference. Strains are mixed in equal amounts and grown to saturation in 96-deep-well plates, where the mixed population ages. (**B**) At regular time intervals, outgrowth cultures are automatically inoculated from the same aging stationary-phase plate onto fresh medium and monitored for growth. The relative initial fraction of viable cells (magenta braces) is approximated from the change in relative fluorescence signal interpolated to a fixed time point (cyan vertical line) in exponential outgrowth. (**C**) The change in ratio of viable cells as a function of age is used to obtain an apparent relative survival coefficient, *s*, which is a direct estimate of the lifespan of *x*Δ relative to WT.

To gain deeper knowledge on which genes shape the lifespan phenotype and—more importantly—on how these genes work together, we developed a sensitive parallel technique for quantifying the CLS of yeast single- or double-gene deletions (Figure 1). We used fluorescent-protein labeling to track the apparent death rates of mutant and wild-type (WT) reference populations in stationary phase. Specifically, each mixed population suspended in stationary phase was automatically sampled at regular age intervals by inoculating to fresh medium, where competition assays were used to estimate the abundance of viable mutants relative to the WT at the time of sampling. An apparent relative lifespan, *L*, of each mutant strain was defined from the change in relative viability as a function of time in stationary phase. Importantly, we accounted for differences in growth rates during outgrowth (see model in Materials and Methods). By using fully-prototrophic strains in all screens, we avoided the strong effects of amino acid auxotrophy on stationary-phase survival [23], [35]. In addition, we buffered the aging medium to identify the lifespan effects of genes and their interactions independently of acetic acid toxicity [36].

We evaluated the performance of our CLS method in several different ways. First, we estimated the technical and biological replicability of our *L* measurements. We observed that the results of two independent large-scale (720 deletion strains) experiments were highly correlated (Pearson's *r* = 0.88; Figure S1). Dye-swap experiments in which 235 deletion strains were independently tagged with both RFP and CFP fluorescent proteins also showed a good correlation (Pearson's *r* = 0.79; Figure S1). In addition, we compared results of our competition-assay with an alternative high-throughput approach that is based on outgrowing individually-cultured strains at different age intervals [20] (Figure S2). Competition-based monitoring of CLS resulted in considerably less variability in the measured phenotypes than experiments in which strains were individually aged and outgrown, underscoring the effectiveness of our experimental approach in terms of precision.

Given that strain-strain interactions could obscure the results of a CLS screen based on competitive survival, we also evaluated the effects of using different reference strains. The relative lifespans of a set of 24 knockouts were mostly consistent across experiments in which different combinations of strains were used (*p*<0.05, Spearman's correlation), indicating that the survival ranks were typically insensitive to the choice of reference strain (Figure S3).

Taken together, these results indicate that our strategy possesses accuracy and robustness that enable the large-scale characterization of CLS at high resolution.

### A high-resolution genome-wide screen for lifespan factors

**Figure 2:**
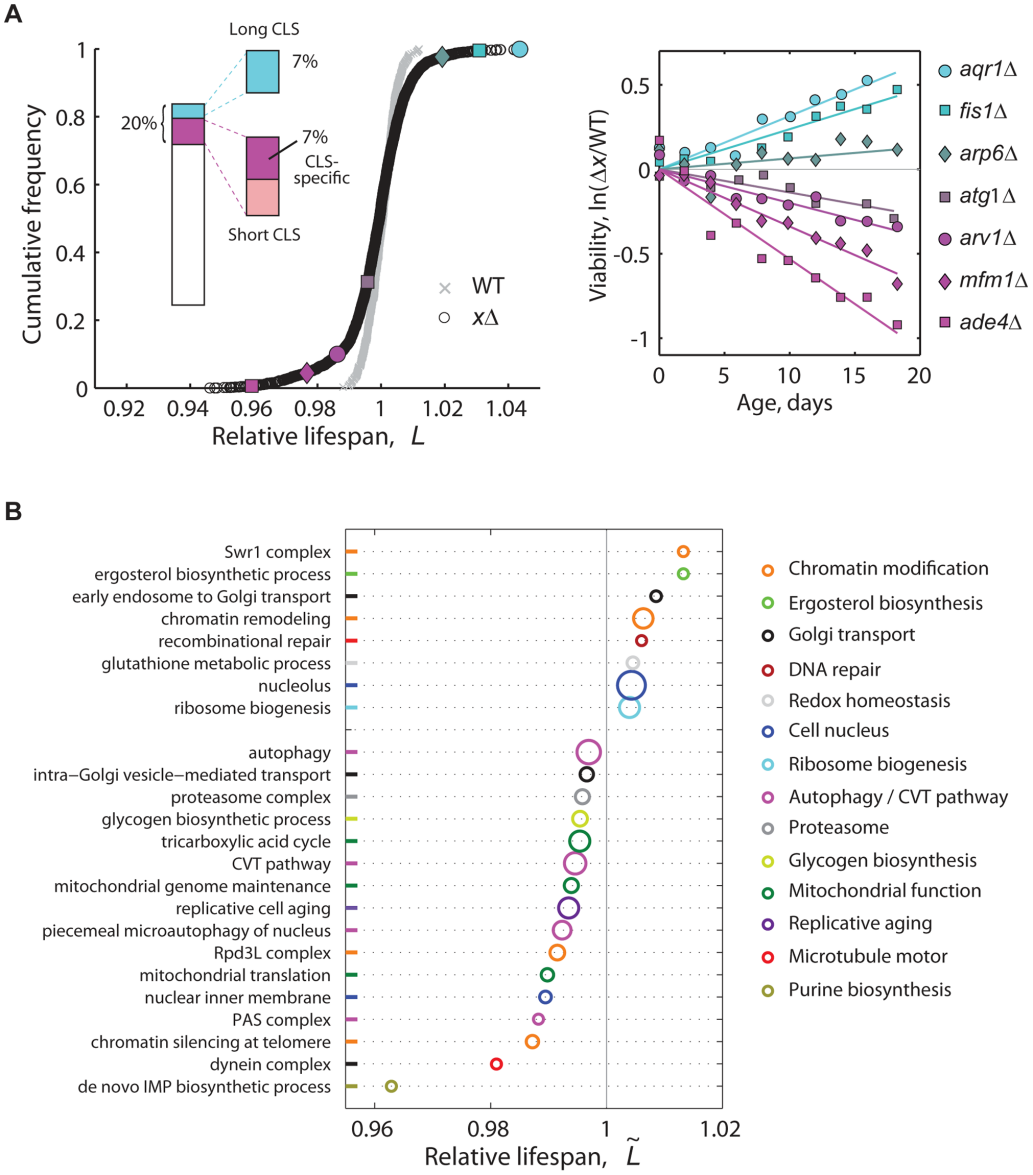
High-resolution genome-wide screening of yeast CLS. (**A**) Cumulative frequencies of the relative lifespan of most viable knockout strains (black circles, *n* = 3,878) and replicates of the WT (gray crosses, *n* = 414). Colored symbols correspond to selected knockouts (right panel). Inset shows the fraction of significant short- (magenta) and long-lived phenotypes (cyan) (5% FDR). Short-CLS phenotypes with reduced growth capacity (*G*<0.95) were filtered-out from subsequent analyses (see Figure S5). (**B**) CLS-specific gene groups and pathways; plot shows the median relative lifespan (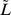) of genes annotated in the same GO term, which was compared in each case to the median lifespan of the entire data set. Labels at the vertical axis show representative enriched GO categories (*p*<0.05, Wilcoxon rank sum test). The radius of each circle indicates the number of measured gene knockouts within the GO group, the minimum being five (*de novo* IMP biosynthesis) and the maximum 39 (nucleolus); colors were arbitrarily assigned to similar functional categories. Data set includes 3,286 strains that have no marked defect in growth (*G*>0.95).

To screen the genome for lifespan-determining genes and pathways, we used our CLS method to characterize 3,878 nonessential-gene knockouts from the yeast deletion collection [37] (data set in Table S1). The distribution of lifespan effects peaked near neutrality (*L* = 1), with a long tail of short-lived knockouts and a smaller but substantial proportion of long-lived strains (Figure 2A). Given that our high-throughput screen was performed at low aeration and not under the conventional aeration conditions (see Materials and Methods), we characterized a subset of mutants at high aeration and confirmed that there was a general agreement in their long- and short-lived behaviors regardless of aeration (Figure S4).

In genetic studies, mutations that increase a maximal lifespan are more likely to be informative about aging processes than mutations that reduce the life span [38], as such deleterious phenotypes can arise from a variety of reasons not necessarily related to aging. Indeed, we observed that short-lived knockouts are more likely to be affected in their rate of exponential growth than strains with wild-type CLS (Figure S5). Still, we note that at least 64% of the short-lived knockouts identified in our genome-wide screen were specific for CLS, that is, their deletion affected stationary-phase survival but not the rate of growth (Figure S5). Furthermore, the overlap of mutants affected in both stationary-phase survival and exponential-growth rate did not increase substantially when a subset of mutant strains were grown non-fermentatively under ethanol-glycerol medium (Figure S6), which indicates that respiratory integrity and its overlap with stationary-phase survival is largely taken into account by describing the mutant's fermento-respiratory growth capacity under standard glucose medium.

We found that an important fraction of the non-essential yeast genome mediates CLS. Overall, 6.8% of single-gene knockouts had a long-lived phenotype, while 7.2% lived significantly less than the wild type without showing major growth defects (5% FDR; see Figure 2a inset, genes in Table S2). To quantitatively describe the cellular processes and protein complexes that underlie CLS in yeast, we also identified functional groups of single deletions with a skewed distribution of lifespan effects (Figure 2B). Mutant strains with compromised mitochondrial function, *de novo* purine biosynthesis, vacuole function, and autophagy typically resulted in reduced lifespan phenotypes. This confirmed the main findings of recent genome-wide screens for yeast CLS factors [21]–[23]. We also note that genes involved in the regulation of replicative cell aging were enriched in CLS effects.

An inspection of the list of short- and long-lived strains (Table S2) indicated that we were also able to confirm a number of previously-reported CLS genes. For instance, inactivation of the Leu3 transcription factor involved in amino acid biosynthesis increased CLS, as expected [39]. Lifespan extension was also observed for *ald6*Δ [40]. In another example, depletion of the apoptotic proteins Fis1 and Aim14 extended yeast CLS, in consonance with studies showing that chronological aging induces apoptosis in yeast [41], [42]. This was also the case of the mitochondrial ubiquinone oxidoreductase: disruption of Ndi1 decreases ROS production and elongates the CLS of yeast [43]. Moreover, strains impaired in Msn2, Glc8, and Glc3 had reduced lifespan, which was in agreement to studies showing that decreased Tor activity activates stress-response transcription factors and genes for glycogen accumulation [19].

Importantly, our screen allowed us to uncover biological processes and protein complexes that had not been previously associated to the CLS phenotype. Noteworthy, depletion of a number of chromatin-modification proteins resulted in extended lifespan (Figure 2B). Such novel chronological aging factors included the Swr1-complex proteins Arp6, Swc3, and Swr1. The SAGA-complex subunits Chd1 and Sgf11 were also, to our knowledge, previously unrecognized pro-aging proteins, which add to proteins of the SAGA-like complex as a histone-modifying factors underlying yeast lifespan [44]. On the other hand, disruption of the Rpd3L histone-deacetylation complex subunits Cti6, Dep1, Rxt2, Rxt3, and Ume1 resulted in short lifespan, without altering the cell's growth capacity. Likewise, the Sdc1, Spp1, and Swd1 subunits of the Set1/COMPASS histone-methylation complex were novel yeast CLS factors (see Table S2). We also identified a number of novel lifespan phenotypes associated to genes with roles on sterol and sphingolipid metabolism and homeostasis (Figure 2B; Table S2). Depletion of the ergosterol-biosynthetic genes Erg5, Erg6, and Hmg1 extended CLS, while disruption of the fatty-acid elongase Sur1—involved in sphingolipid biosynthesis—reduced CLS without affecting growth. The *ARV1*-encoded protein, which mediates sterol and shingolipid homeostasis [45]–[48], was also a novel lifespan factor identified in our genome-wide CLS screen.

### Confirming chronological lifespan phenotypes and estimating extents of adaptive regrowth

**Figure 3:**
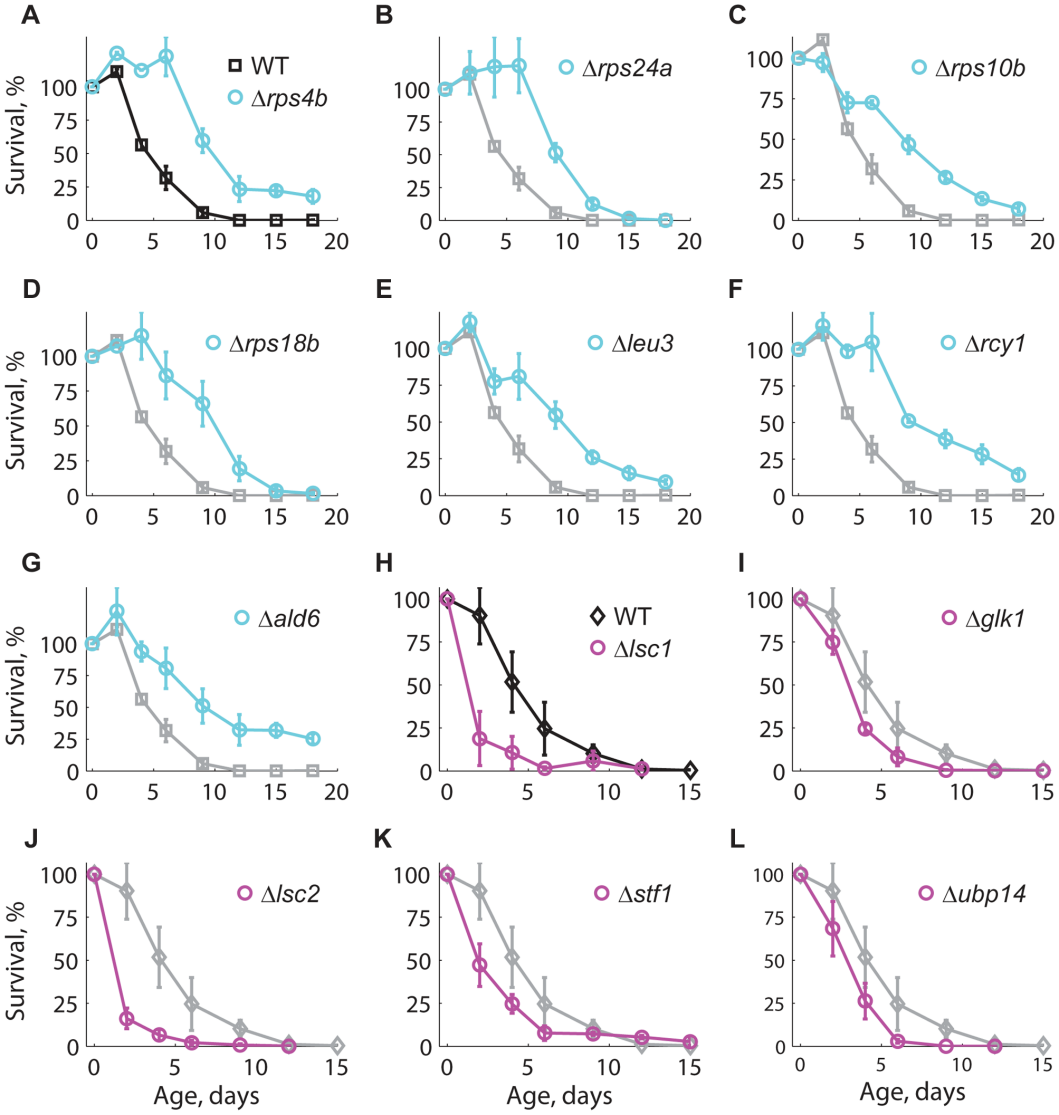
Longevity phenotypes were confirmed using the standard CLS method. Survival curves under non-buffered SC medium for WT (black), (**A–G**) long-lived (cyan), and (**H–L**) short-lived knockout strains (magenta). Only those strains that were different from the WT are shown; for comparison, the CLS of the WT is shown in each panel (gray). Each strain was assayed in three independent experiments; data points are the average and error bars indicate the SEM. One-hundred percent survival corresponds to the colony-forming units measured at day four after inoculation (age zero).

To further validate the results of our competition-based genome-wide screen, we assayed 14 of the longest-lived and seven of the shortest-lived knockout strains using the standard CLS method with non-buffered medium [49] (see Materials and Methods). Knockout strains Δ*rps4b*, Δ*rps24a*, Δ*rps10b*, Δ*rps18b*, Δ*leu3*, Δ*rcy1*, and Δ*ald6* were confirmed as long-lived (Figure 3A–G), while Δ*lsc1*, Δ*glk1*, Δ*lsc2*, Δ*stf1*, and Δ*ubp14* were confirmed as short-lived (Figure 3H–L). The CLS of strains deleted for *ADE5,7*, *AQR1*, *CYB5*, *ERG6*, *NEW1*, *RRP8*, *SPP1*, *YAK1*, and *YPL062W* was not distinguishable from the WT when retested (data not shown). Overall, seven out of 14 long-lived strains and five out of seven short-lived knockouts were confirmed using the standard CLS method.

Subpopulations in aging stationary phase cultures may undergo adaptive regrowth, a phenomenon whereby non-dividing cells acquire mutations that allow them to re-enter the cell cycle and take advantage of the nutrients released by dead cells [50]. If the rate of adaptive regrowth of a given mutant strain was different than the wild-type, such mutant would register an artificially high or low abundance ratio as a function of age. We therefore estimated the extent at which adaptive regrowth gave rise to false-positive lifespan factors in our screen (Figure S7). To do so, we focused on nearly all mutant strains scored as short- or long-lived (505 out of 511 knockouts; see Table S2) and monitored their chronological aging in water to prevent the occurrence of adaptive regrowth [49]. We observed that the phenotypes from these two aging conditions were consistent with each other; the distributions of lifespan effects in water were markedly different between mutants scored as short-lived or long-lived in buffered medium (*p*<10^−79^, Wilcoxon rank sum test). In other words, strains that aged fast or slow in buffered medium typically did so in water as well. For instance, the CLS of only one of the longest-lived and one of the shortest-lived strains in buffered medium were not significantly different from the WT CLS in water (see Table 1). Overall, we were able to confirm 61% short-lived and 48% long-lived phenotypes, which sets such minimal fractions of true-positive lifespan factors in our original genome-wide screen in terms of accounting for adaptive regrowth.

**Table 1:** Chronological-lifespan effects of strains aged in SDC medium and in water.

	SDC buffered medium		Water	
Strain	Relative lifespan, *L* a	*p*-value^b^	Relative lifespan, *L* a	*p*-value^b^
WT	1		1	
**Long-lived strains**				
*rps4b*Δ	1.033±0.003	<10^−3^	1.009±0.010	0.105
*rps24a*Δ	1.035±0.007	<10^−3^	1.014±0.009	0.045
*rps10b*Δ	1.042±0.004	<10^−3^	1.020±0009	0.006
*rps18b*Δ	1.035±0.003	<10^−3^	1.014±0.014	0.043
*leu3*Δ	1.012±0.004	0.003	1.015±0.006	0.032
*rcy1*Δ	1.062±0.006	<10^−3^	1.051±0.005	<10^−3^
*ald6*Δ	1.109±0.005	<10^−3^	1.063±0.005	<10^−3^
**Short-lived strains**				
*lsc1*Δ	0.964±0.003	<10^−3^	0.975±0.009	<10^−3^
*glk1*Δ	0.957±0.003	<10^−3^	0.999±0.015	0.444
*lsc2*Δ	0.964±0.002	<10^−3^	0.984±0.006	0.022
*stf1*Δ	0.959±0.003	<10^−3^	0.973±0.007	<10^−3^
*ubp14*Δ	0.963±0.004	<10^−3^	0.971±0.010	<10^−3^

a Relative lifespan is obtained from the survival coefficient (1+*s*), expressed as the change in viability per day relative to the WT reference, ± error in the linear fit.

b *p*-value is from the Z-score of 414 or 65 WT replicates in SDC medium or in water, respectively.

### The Swr1 complex is a novel aging factor that mediates lifespan extension by dietary restriction

**Figure 4:**
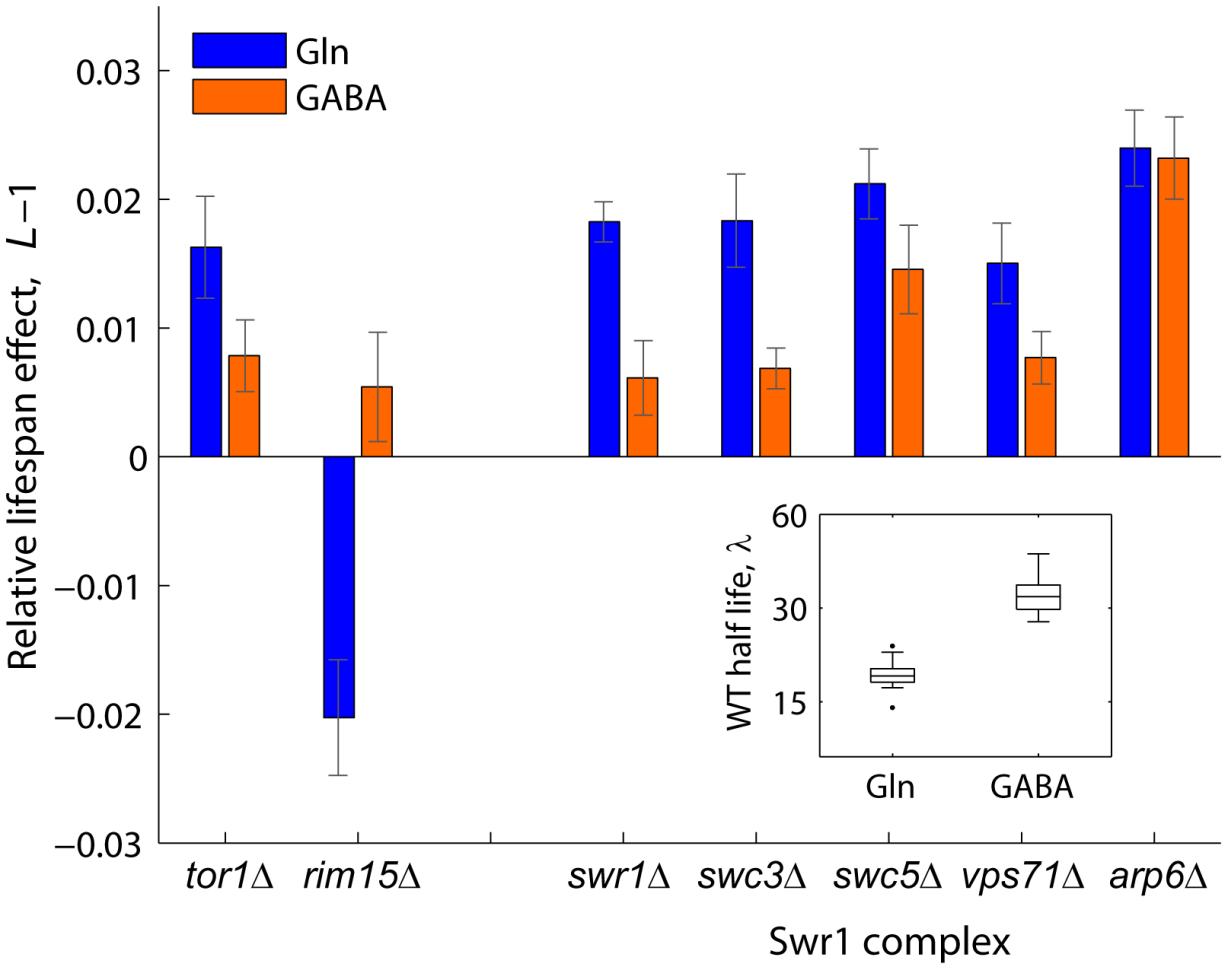
Relative lifespan extension by inactivation of Swr1 is partially abolished under dietary restriction. Bars show the relative lifespan effects (*L*−1) for mutant strains aged in competition against the WT in glutamine (rich nitrogen, blue bars) or GABA (poor nitrogen, orange bars) aging medium. Error bars indicate the standard error from at least three independent experiments. Box plot (inset) shows the absolute half life measurements of individual WT cultures aged under glutamine or GABA (*n* = 28).

Swr1 is a multisubunit chromatin-remodeling complex that contains the *SWR1*-encoded Swi2/Snf2-related ATPase and is required for the incorporation of the histone variant H2A.Z into chromatin [51]. To further investigate the mechanisms by which activity of the Swr1 complex promotes chronological aging, we quantified the lifespan effects of Swr1 mutants under glutamine (nitrogen-rich) or GABA (nitrogen-poor) conditions (Figure 4). By doing so, we asked whether lifespan extension by dietary restriction depends on the reduced activity of this novel aging factor. Interestingly, the Swr1-complex gene knockouts lived relatively longer than the WT in glutamine, but not under nitrogen-limited condition, with the exception of *arp1*Δ which had a significant effect in both GABA and glutamine (Figure 4). As a control, we analyzed the effects Tor1 and Rim15, which are known to be key players of the pathway that extends longevity by nutrient limitation [13]. As expected, the *tor1*Δ and *rim15*Δ strains showed, respectively, increased and decreased relative longevity preferentially under nitrogen-rich conditions. These results suggest that lifespan extension by dietary restriction depends, at least in part, on the action of the Swr1 histone-exchange complex.

### A functional link between Arv1 and autophagy underlies yeast lifespan

**Figure 5:**
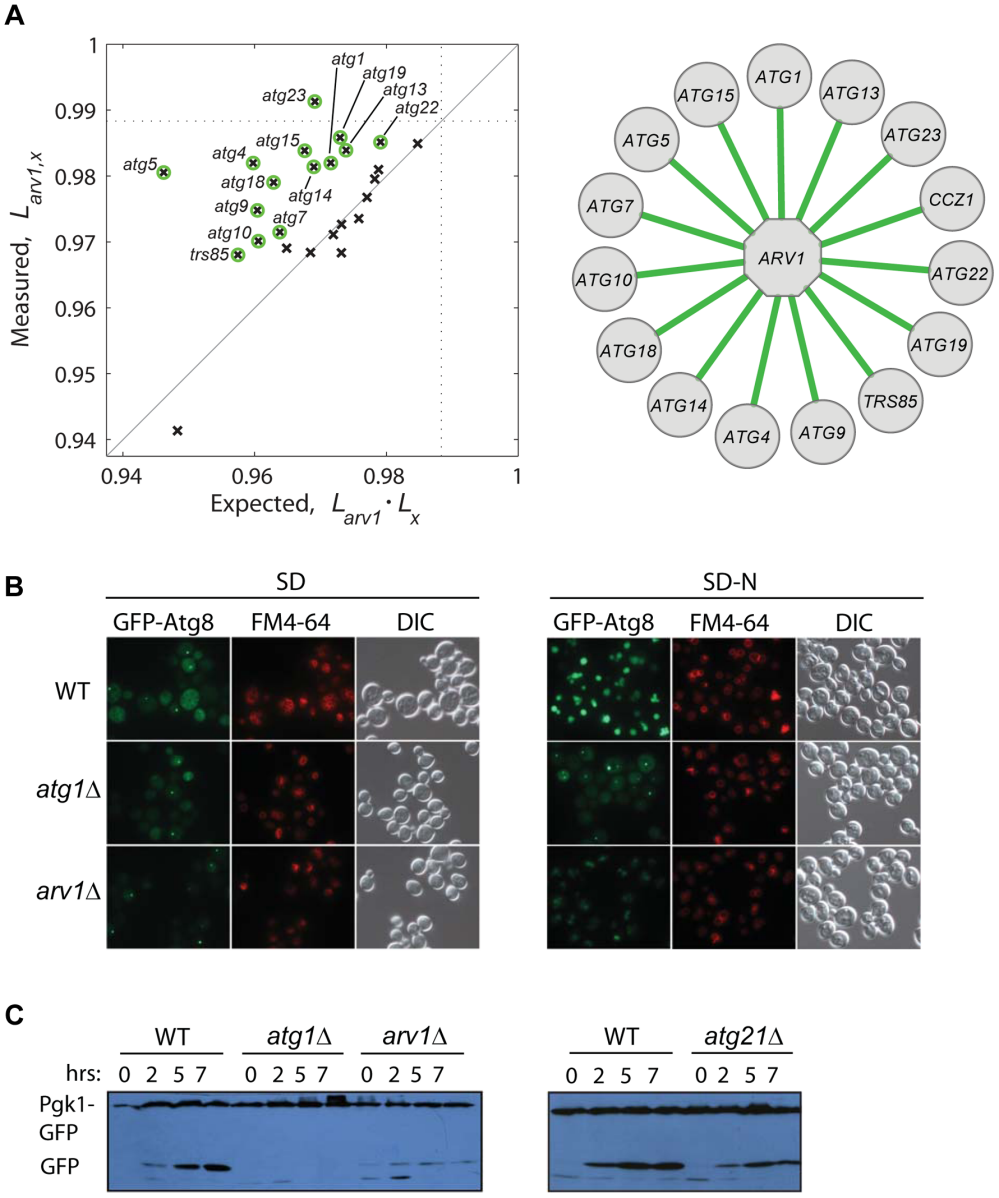
Functional link between autophagy and Arv1 underlies CLS. (**A**) Genetic interaction analysis of *ARV1*. The relative lifespan of 26 *arv1*Δ, *x*Δ double knockouts is plotted against the expected multiplicative combination of the corresponding pairs of single knockouts. Dotted lines at the horizontal and vertical axes show the effect of the *arv1*Δ single knockout. Green circles indicate significant deviations from the neutral expectation (>95% CI), which are shown as green edges connecting *ARV1* to the interacting gene (right). (**B**) Micrographs of WT and gene-knockout strains expressing the GFP-Atg8 fusion protein under basal conditions (SD, left) and after 7 h of nitrogen starvation (SD-N, right). Vacuoles were stained with FM4-64; DIC images show the position of yeast cells. (**C**) Western blot analysis of nitrogen-starved strains expressing the fusion protein Pgk1-GFP: WT and cells depleted for Arv1, the core-autophagy Atg1 protein, or the accessory Atg21 protein. Time zero corresponds to mid-log cultures before transfer to SD-N medium where cells grew for 2, 5, or 7 h before lysis.

To gain deeper knowledge on the lifespan role of Arv1, a protein involved in lipid homeostasis, we asked whether this novel CLS factor modulates yeast lifespan together with or independently from autophagy, which is a conserved modulator of lifespan in yeast and multicellular eukaryotes [14], [19], [52] and is intimately linked to lipid metabolism [53]. We generated a collection of double knockouts combining *arv1*Δ to 26 deletions of core-autophagy or CVT-pathway genes. Strikingly, the relative lifespan of many double knockouts was significantly increased when compared to the expected from the independent combination of the deleterious lifespan phenotypes of each of the corresponding single knockouts (Figure 5A). This result suggested that the anti-aging effect of autophagy depends to some extent on the action of Arv1.

To confirm the observed genetic association of Arv1 with the autophagy machinery, we monitored different reporters of autophagic activity in wild-type and Arv1-disrupted cells. We found that *arv1*Δ cells accumulate less Atg8—a marker of autophagic bodies—in the vacuole, partially resembling the molecular phenotype of *atg1*Δ cells in which autophagy activity is completely blocked (Figure 5B). In addition, we examined the degradation of Pgk1-GFP, a low-turnover rate cytosolic fusion protein that is rapidly degraded by non-selective autophagy upon nitrogen starvation [54]. Also in agreement with the genetic interactions scored between autophagy genes and *ARV1*, we found that the autophagy-directed appearance of processed GFP protein was clearly diminished in *arv1*Δ cells (Figure 5C). Taken together, these results showed that autophagic activity was compromised in *arv1*Δ deletion strains and confirmed a novel connection of Arv1 with the autophagy pathway, as anticipated by means of genetic- interaction analysis.

### Deriving genetic interactions systematically from quantitative lifespan phenotypes

The combination of high throughput and sensitivity of our phenotyping approach not only provides an accurate catalogue of genes and pathways underlying CLS, but also lends itself for the systematic construction of lifespan-based quantitative genetic interaction (GI) maps. By describing the ways in which mutations affect each other's phenotypes, GI analyses in yeast have been very successful in elucidating genetic pathways [27]–[31], [55]. Since cell-proliferation phenotypes are typically used to generate such maps, and given that the genes that promote growth are not necessarily important for survival, it was expected that CLS-based GI data would uncover novel pathways, with particular relevance to aging. Indeed, we noted that 64% of the genetic factors identified in our genome-wide screen were specific for stationary-phase survival, that is, their deletion affected CLS but not the rate of exponential colony growth (Figure S5).

For the systematic study of lifespan-epistatic interactions we focused on the autophagy pathway, which drives the degradation and recycling of unnecessary or dysfunctional cellular components [56]–[58]. While autophagy has consistently been shown to modulate CLS, the way in which autophagy genes interact phenotypically with one another and with genes of related processes has not been systematically analyzed. We surveyed interactions for almost all core-autophagy genes, along with several signaling and accessory factors (39 genes and all possible combinations). The single knockout of most genes examined had at least a small but significant deleterious effect on CLS, and the measured CLS phenotypes of two marker-swap experiments showed a good correlation (Figure S8). Importantly, to verify the CLS specificity of the genes tested, we confirmed that exponential growth capacity of their single knockouts remained largely unaffected (Figure S9).

**Figure 6:**
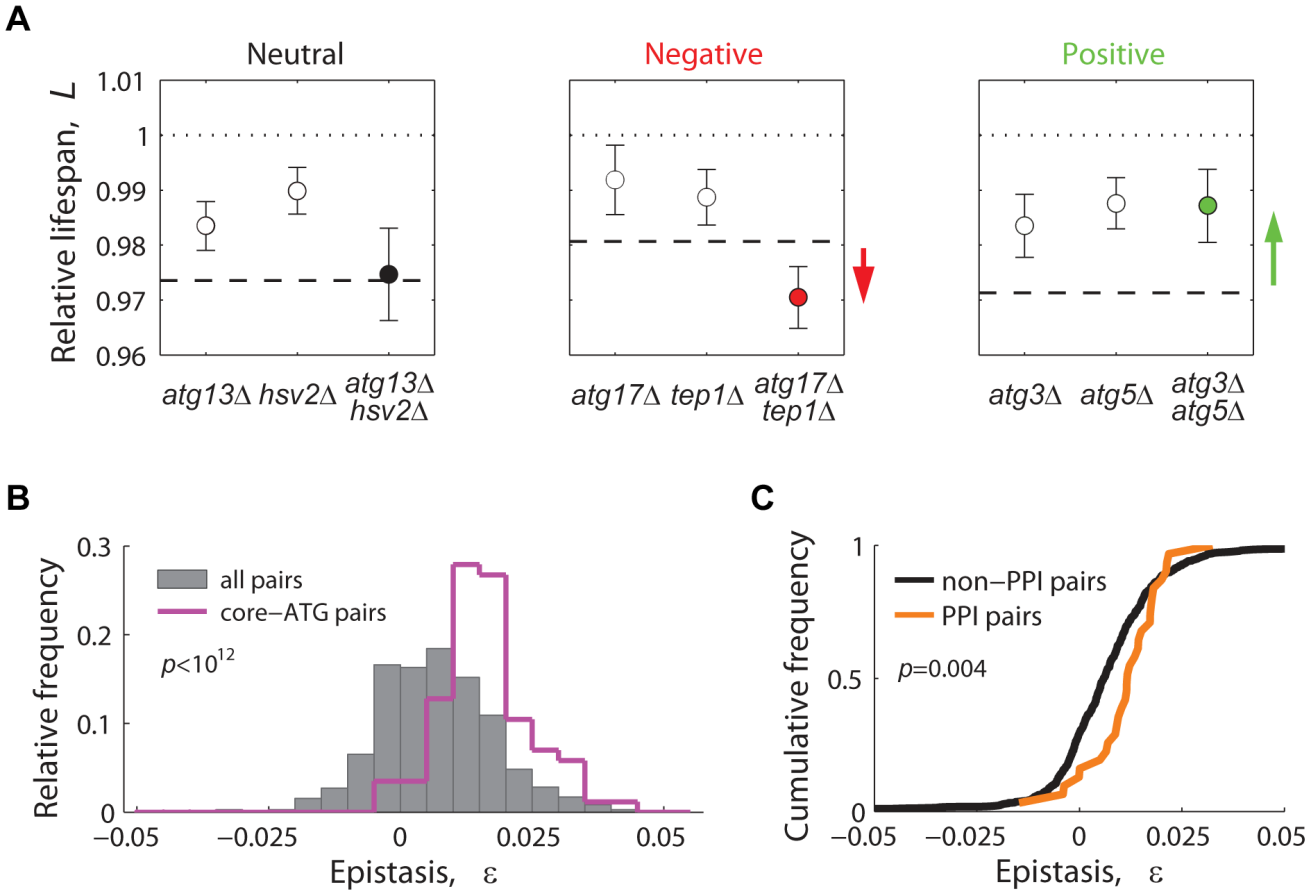
Lifespan-epistatic interactions recapitulate functional and physical associations. (**A**) Plots show the measured relative lifespan of representative gene pairs (single and double knockouts) with neutral (left panel), negative (center panel), and positive epistasis (right panel). Dashed lines indicate the multiplicative expectation in each case, that is, *L_x_*_Δ_ · *L_y_*_Δ_. Error bars indicate the 95% CI. (**B**) Spectrum of epistasis measured for all gene pairs (gray histograms, *n* = 721) and for core-autophagy gene pairs (purple histogram outline, *n* = 75); (**C**) Comparative cumulative distributions of ε for gene pairs coding for proteins that interact (orange line, *n* = 31) or do not interact physically (black line, *n* = 690); the *p*-value is from the Wilcoxon rank-sum test.

For each gene pair, lifespan epistatic interactions were defined as significant deviations from a null multiplicative expectation based on the CLS phenotype of the two single knockouts. Neutrality was defined when the lifespan phenotypes of the single knockouts combined multiplicatively, suggesting that the two genes act independently from one another (Figure 6A, left panel). Departing from this neutral expectation, GIs were classified as either negative (aggravating) or positive (alleviating) [59]: Negative epistasis was scored when the double mutant exhibited a significantly shorter CLS phenotype than one would expect from the CLS of the two single knockouts (Figure 6A, center panel). Such aggravating phenotype is expected from the deletion of pairs of genes that act in lifespan pathways that partially compensate for each other's loss. Conversely, in a positive epistatic interaction, the double mutant exhibited an extended CLS relative to the neutral expectation (Figure 6A, right panel). In general, this phenotypic response takes place when both deleted genes are part of the same linear lifespan-determining pathway or protein complex.

Overall, we scored a significant GI for 201 out of 721 gene pairs tested (3.2% negative and 24.7% positive, 95% CI, see data in Table S3). In agreement with the common assumption that most gene pairs do not interact, we found that the lifespan-epistasis spectrum peaked close to neutrality, though skewed to positive epistasis (gray histogram in Figure 6B). Many of the positive interactions in our data set corresponded to gene pairs within the core-autophagy machinery (purple histogram outline in Figure 6B). For this set, we scored 55.8% significant positive interactions and no negatively interacting gene pair. The distribution of epistatic effects remained largely unaffected when interactions were defined from an additive rather than multiplicative neutral expectation model (Figure S10).

We also observed that the distribution of epistasis among gene pairs that code for proteins that interact physically had a higher median ε value than that of gene pairs whose protein products do not interact (Figure 6C). In summary, the observed prevalence of positive epistasis within our data set was consistent with the fact that many autophagy proteins operate in concert as a non-essential functional unit that promotes CLS.

### A lifespan-epistasis map for autophagy

**Figure 7:**
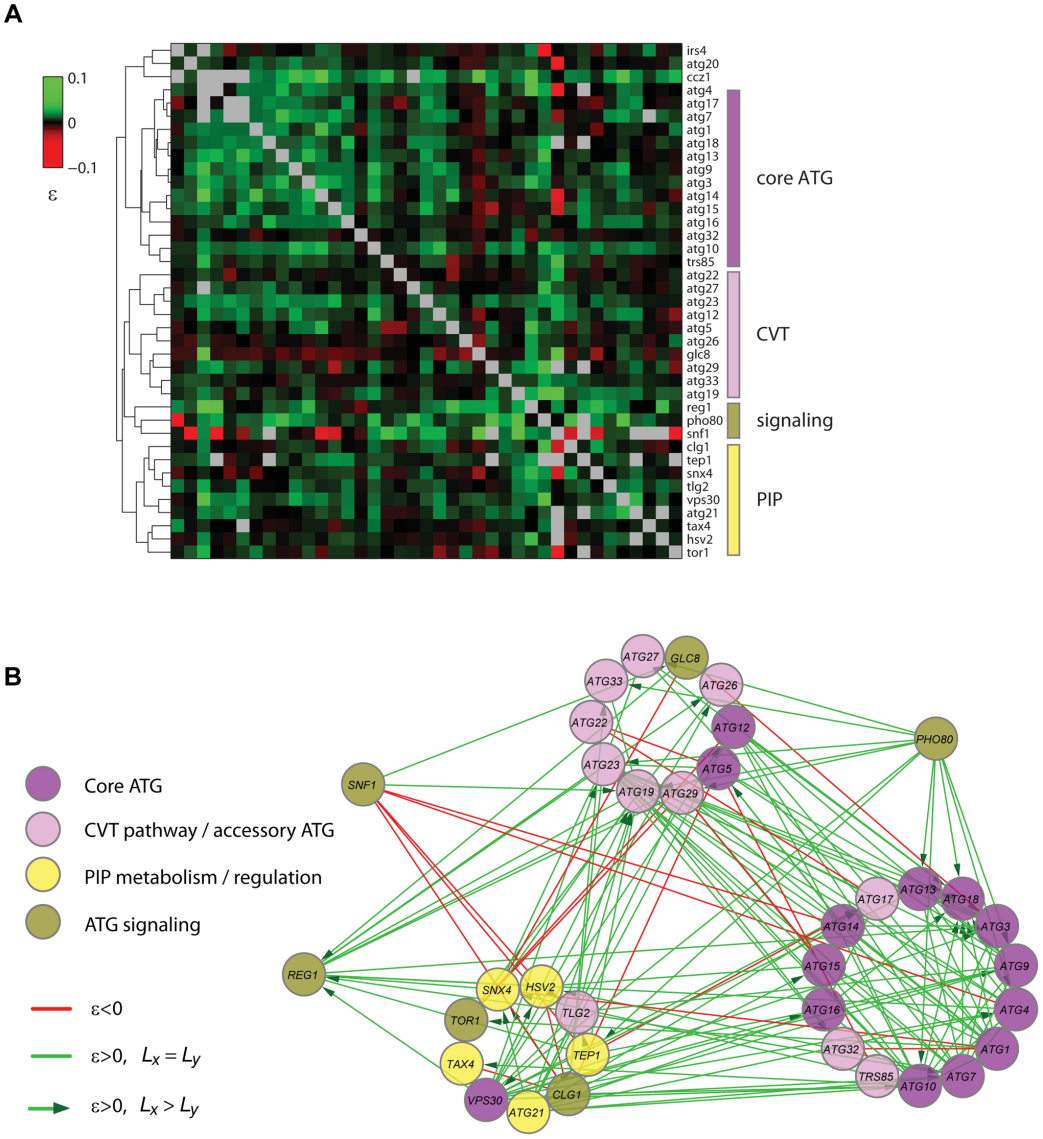
Quantitative lifespan-epistasis analysis reveals functional associations within and between pathways underlying CLS. (**A**) Hierarchical-cluster analysis of lifespan-epistasis data. Each column/row represents the interaction pattern for a specific gene; the color scale indicates the epistasis interaction strength, ε. Dendrogram clusters are highlighted in colored bars (right). (**B**) Network representation of gene clusters and their interactions. Node color depicts the gene's known biological function. Edges show significant negative (red) or positive (green) epistasis (95% CI). Arrows indicate cases of significant CLS-differences between the two single knockouts, with directionality from the largest to the smallest single knockout effect.

Based on the correlation of the quantitative epistatic-interaction pattern derived from CLS phenotypes of each gene, we constructed a high-density GI map for autophagy (Figure 7A). Using purely phenotypic information, this unsupervised map recapitulated known pathways and complexes and shed light on novel gene associations. With few exceptions (Atg5, Atg12, and Vps30), all core autophagy proteins were grouped together (core-ATG module, Figure 7A). Proteins with a major role in the CVT pathway (Atg19, Atg23, and Atg27) were clustered together with other autophagy-accessory proteins and apart from the core-ATG module (CVT module, Figure 7A).

We also used gene-clustering information along with gene-interaction significance to generate a lifespan-epistasis network (Figure 7B). We observed a high enrichment of positive epistatic interactions among genes within the core-ATG module, again consistent with the fact that core-autophagy proteins act as a single functional unit (*p*<10^−4^, Fisher's exact test). Furthermore, directionality was assigned to positive epistatic interactions when one of the genes had a larger single-knockout effect than its interaction partner [30]. For instance, we scored positive interactions targeted to *ATG18*; single-deletion of this gene had a smaller quantitative effect on CLS when compared to the typical core-autophagy gene. This observation was consistent with the fact that Atg18 is only required for reaching full autophagic activity and most autophagy proteins localize to the pre-autophagosome in the absence of Atg18, but not in the absence of other core-autophagy proteins [60].

Our lifespan-epistasis map also recapitulated a group of proteins related to phosphatidylinositol phosphate metabolism and regulation (Figure 7B), including Atg21, Tax4, Snx4, Hsv2, and Tep1. The association of Tep1 within the PIP module provided genetic evidence for its putative role as a phosphoinositide phosphatase. In cancer cells, the *TEP1* homologue *PTEN* controls autophagy by downregulating the PI3KclassI/PKB pathway [61]. The syntaxin-like t-SNARE Tlg2 protein was also part of this PIP module. Recent evidence suggests that vesicle-tethering proteins and some SNARE proteins are required for autophagosome biogenesis and normal anterograde transport of Atg9 [62]. This result provided further genetic evidence for the role of Tlg2 as a SNARE protein which is essential for autophagy and suggested that this functional association is needed for normal lifespan.

Three signaling factors (Pho80, Snf1, and Reg1) were grouped together in our lifespan-epistasis map (Figure 7A,B). For *PHO80*, positive epistatic interactions were all directed from this signaling gene to autophagy genes, in agreement with the regulatory role of Pho80-Pho85 upstream of autophagy [63] and suggesting that this cyclin regulates other downstream pathways that are important for yeast CLS. As opposed to *PHO80*, *SNF1* interacted negatively with the core-ATG and PIP modules, which may reflect that Snf1 independently promotes autophagy and inositol biosynthesis [64], [65].

Taken together, these results show that quantitative lifespan-epistasis maps represent a powerful systematic strategy to establish functional associations within and between aging pathways.

## Discussion

We have used a novel functional genomics platform to generate a high-resolution compendium of genes that affect yeast stationary-phase survival and to construct the first systematic GI map based on lifespan phenotypes.

Our genome-wide screen indicated that a substantial fraction of the non-essential yeast genome (14%) regulates stationary-phase survival independently of general growth defects. Among novel CLS factors detected in our screen were different proteins of the Swr1 complex, which is required for the ATP-dependent recruitment of histone variant Htz1—the yeast homologue of mammalian H2A.Z—and promotes expression near silent heterochromatin [51]. It is known that chromatin structure and dynamics change during the aging process [66]. To further explore the specific role of the Swr1 complex as a regulator of CLS in yeast, we characterized a number of Swr1-complex knockouts under different nitrogen conditions. In doing so, we provided evidence suggesting that lifespan extension by dietary restriction depends on the activity of the Swr1 complex.

We also showed that Arv1—an endoplasmic reticulum protein conserved from yeast to mammals—and autophagy promote chronological longevity in concert. Such genetic association of *ARV1* was confirmed by monitoring different autophagy reporters in Arv1-depleted cells. This result adds to a growing body of evidence suggesting that autophagy is intimately connected to lipid metabolism: While autophagy regulates lipid metabolism [53], sphingolipids and ceramides play important roles in autophagy itself, both at the level of regulation and as membrane constituents of autophagic vesicles [67], [68]. In the absence of Arv1, yeast cells up-regulate the unfolded protein response [69] and display a variety of lipid-related phenotypes that include aberrant sterol and sphingolipid metabolism and loss of plasma-membrane asymmetry [45]–[48]. In humans, Arv1 is required for normal cholesterol and bile acid homeostasis [70]. In regard to our finding that autophagy activity depends on Arv1, we speculate that this protein could be specifically required for proper targeting of Trs85 to the pre-autophagosomal structure, as it has been recently shown that Arv1 is involved in the targeting of tail-anchored proteins to the endoplasmic reticulum [48]. Alternatively, the general lipid-homeostasis role of Arv1 could be essential for the correct activity of the autophagy machinery. In any case, our results indicate that the functional link between Arv1 and autophagy has an impact on cellular lifespan.

Our systematic lifespan-epistasis network successfully recapitulated functional associations of the autophagic machinery and related biological processes. While most core-autophagy genes clustered together as expected, few exceptions included *ATG5* and *ATG12*, which were grouped with genes of selective autophagy and of the CVT pathway. A specific role of *ATG5* and *ATG12* has been reported for mitophagy and lifespan regulation in human umbilical vein endothelial cells: Mitochondrial damage induces upregulation of these genes together with *LC3B*/*ATG8*, which in turn increases the replicative cellular lifespan [71]. Furthermore, our epistasis network established that Tep1 and Tlg2 work in concert with the phosphatidylinositol-phosphate metabolism pathway to modulate lifespan. While it has been shown that Tep1 is not a replicative lifespan factor in yeast [72], our data indicated that Tep1 activity promotes CLS, in line with *PTEN*-overexpressing mice with increased longevity [73]. The CLS phenotype of yeast Tep1 and its association to other phosphatidylinositol proteins provide a simple model system to elucidate the molecular mechanisms underlying the role of its mammalian homologue *PTEN* in embryonic survival and tumor suppression [74].

Compared to previous genome-wide screens in which 6% [19] or 31% [21] of longevity phenotypes were confirmed when retested, our approach performed modestly better by correctly predicting 50% (seven out of 14) knockout strains with extended lifespan. There are a number of possible reasons why not all of the mutants retested with the standard CLS assay were successfully confirmed. Our relative lifespan measurements are based on a simple deterministic model that assumes constant exponential death and outgrowth rates as the population ages. For instance, cell heterogeneity of stationary-phase cultures [75] was not possibly taken into account. Moreover, results of our control screens suggest that differences in aeration or the effects of adaptive regrowth explain part of the false positives that were obtained. Importantly, once a set of mutants have been confirmed, our approach can be readily used to leverage the chronological aging paradigm to generate aging networks, as we have shown for autophagy. Our pure phenotypic characterizations proved powerful for establishing functional relationships among such genes that had remained hidden to large-scale genetic analysis. Despite the fact that our epistasis data set is limited in its scale and is *a priori* enriched for genes that share functional relationships, we anticipate that the strategy herein described will open the door to describe the spectrum of lifespan-epistatic interactions in a comprehensive and unbiased manner.

With a growing number of lifespan-related genes, a systems view of aging is much needed to grant a deeper mechanistic understanding of cellular longevity [7], [76]. Our quantitative analysis of longevity factors and their genetic interactions in yeast is an important step to this end.

## Materials and Methods

### Strains and media

Fluorescent SGA starter strains YEG01-CFP and YEG01-RFP (*MATα PDC1-XFP-CaURA3MX4 can1*Δ::*STE2pr-SpHIS5 lyp1Δ his3*Δ*1 ura3*Δ*0 LEU2*) were generated by direct PCR-based tagging of the S288C-derivative SGA starter strain Y8205 [77] with the pBS10 Cerulean-*hphMX4* (CFP) or pBS35 mCherry-*hphMX4* (RFP) cassettes (The Yeast Resource Center) at the *PDC1* locus and subsequent direct replacement of the *hphMX4* cassette with *CaURA3MX4*; strong constitutive expression of fluorescent proteins during exponential growth was achieved by carboxyl-terminal fusion to the Pdc1 protein. Deletion strains were from the yeast deletion collection (Open Biosystems) in the S288C-derivative BY4741 background (*MATa xxx*Δ::*kanMX4 his3*Δ*1 ura3*Δ*0 leu2*Δ*0 met15*Δ*0*). The *GFP-ATG8* fusion was generated by PCR-based tagging with the *natMX4-CUP1pr-yeGFP* cassette from pYM-N4; the *PGK1-GFP* fusion was obtained from the GFP-tagged yeast strain collection (Invitrogen). Genes of interest were knocked-out from GFP fusion strains by direct gene replacement with the *kanMX4* cassette.

The following growth media were used: (1) Aging medium: synthetic complete (SC) medium with 2% glucose, 0.2% amino acid supplement mix as defined (Cold Spring Harbor Laboratory Manual 2005), and buffered to pH 6.0 with 17.9 mM citric acid and 64.2 mM dibasic sodium phosphate. When noted, 25 mM glutamine or 25 mM gamma-aminobutyric acid (GABA) instead of ammonium sulfate and 0.07% of amino acid supplemented mix were used. (2) Low-fluorescence medium (YNB-lf) [78]. (3) Nitrogen-starvation medium (SD-N): 0.17% yeast nitrogen base without amino acids and ammonium sulfate and with 2% glucose. (4) Yeast-extract peptone with 2% dextrose (YPD) or 2% ethanol, 2% glycerol (YPEG) medium.

### Generation of yeast libraries

Collections of prototrophic yeast strains were generated by synthetic genetic array (SGA) methodology [77]. Colony arrays were transferred manually with a 384-head pin tool (V&P Scientific, VP384F); antibiotic concentrations used for selection were 200 µg/ml G418 (Invitrogen) and 100 µg/ml clonNAT (Werner BioAgents).

To generate the collection of RFP-tagged strains used for the genome-wide CLS screen, the YEG01-RFP starter strain was mated to an array of 4,844 viable single-deletion strains, followed by diploid selection, sporulation, and three rounds of haploid selection (*HIS^+^* for *MATa* mating type, *URA^+^* for fluorescence marker, and *G418^+^* for knockout selection). The resulting prototrophic strains were *MAT*a *PDC1-RFP-CaURA3MX4 can1*Δ::*STE2pr-SpHIS5 lyp1Δ ura3*Δ*0 his3*Δ*1 LEU2 MET15 xxx*Δ::*kanMX4*.

For the double-knockout collection of autophagy-related genes, 41 query strains were generated by PCR-based gene replacement of the YEG01-RFP background using the *natMX4* module. This query collection was mated to an array of 44 deletion strains. Double-knockout haploid strains were selected as described above, plus clonNAT^+^ for the second knockout. The query collection included two neutral-insertion *ho*Δ controls, while four *his3*Δ controls were present in the array collection. All combinations of a knockout strain with a neutral insertion (up to six replicates) define the “single knockouts”, while the combination of two neutral markers define the “WT strain”. Likewise, the CFP-labeled reference strain was obtained from the YEG01-CFP starter strain by the same procedure of combining two neutral markers. All query and array strains were PCR-verified for correct marker insertion and disruption of the WT allele (data not shown).

To obtain *ARV1* double knockouts, *arv1*Δ::*natMX4* and *ho*Δ neutral-insertion query strains (YEG01-RFP background) were mated in triplicate to an array of 29 autophagy-gene deletion strains, including the *arv1*Δ::*kanMX4* and the *his3*Δ::*kanMX4* neutral-insertion. Double knockouts were selected as described above.

### Automated CLS assay: Experimental setup, robotic integration, and data acquisition

Saturated cultures of RFP-labeled mutant (single or double knockout) or CFP-labeled reference strains grown in SC medium were mixed in a 1∶1 ratio to a final volume of 150 µl in 96-well plates (Corning 3585) and pinned with a manual replicator (V&P Scientific, VP 407) onto 700 µl of fresh aging medium in deep-well plates (Nunc 260251) with disposable plastic covers. All subsequent steps were carried out in an automated robotic station (Tecan Freedom EVO200) that integrates a plate carrousel (Liconic STX110), a high-performance multilabel plate reader (Tecan Infinite M1000), a 96-channel pipetting head, an orbital plate shaker, and a robotic manipulator arm, all contained in an isolated environmental room. Mixed stationary-phase cultures were maintained at constant temperature (30°C) and relative humidity (70%) without shaking; cultures were fully resuspended every 24 h by automated pipetting.

Five days after inoculation (age zero, as previously defined [50]) and every 2–3 days for up to 23 days from this point, stationary-phase cultures were transferred to the liquid-handling deck for outgrowth: 5 µl aliquots were inoculated into 150 µl of fresh low-fluorescence medium in 96-well plates. Such outgrowth cultures were kept at 30°C without shaking and monitored by the following procedure: Every 3 h (15 h, five data points), outgrowth cultures were vigorously shaken and transferred to the plate reader to collect data for both fluorescence channels (*CFPraw*: Ex 433 nm/5 nm and Em 475 nm/5 nm; *RFPraw*: Ex 587 nm/5 nm and Em 610 nm/5 nm) and optical density at 600 m (*OD_600_*). Background fluorescence signal as a function of *OD_600_* was routinely collected from RFP-only (*CFPbg*) and CFP-only (*RFPbg*) control cultures. Fluorescent signal in the outgrowth culture was defined as: *RFP* = *RFPraw*–*RFPbg* and *CFP* = *CFPraw*–*CFPbg*.

### Data analysis

The log of the ratio of *RFP* to *CFP* signal (*lnR/C*) was obtained and a single value for each outgrowth culture at age *T* was calculated by interpolating *lnR/C* to a fixed point in time, t* (see Model below). For all experiments, t* was fixed at 10 h. Importantly, results were insensitive to the choice of t* for outgrowth data collected during late exponential growth (8–12 h post inoculation, data not shown). An apparent survival coefficient, *s*, and its standard error, σ*_s_*, were obtained from the slope of the linear fit of lnR/C^t*^ to *T* (robust regression, Matlab). From an empirically determined dynamic range (Figure S11), we defined conservative low and up lnR/Ct* thresholds of −3.5 and 2.5, respectively. Only fits with at least three data points were considered to obtain a value of *s*. Hence, mutant strains with low fluorescence signal or strains that grew too slow in co-culture (less than 5% of the tagged deletion collection) were excluded from our analyses.

All screens included a large number of WT samples scattered through the strain arrays (typically >300 wells); these were used to account both for survival differences between WT and CFP-labeled reference strain and potential batch effects. By definition, the survival coefficient of the RFP-labeled WT strain, 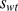, equals zero. Hence, data was normalized by subtracting the median value of all WT control samples, 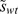, to all raw *s* values in each batch (see example in Figure S12). The mutant's apparent relative lifespan was expressed as the decrease in mutant population relative to WT, that is, 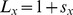.

### Model for outgrowth and stationary phase populations in co-culture

In our experimental setup, outgrowth from stationary phase is used to estimate the relative numbers of viable cells across an experiment. In practice, this quantity is obtained after several hours of inoculation into fresh medium. In what follows, we show that measuring the ratios of two exponentially-growing populations at a fixed time-point in hours, t*, across different ages of the culture in stationary phase, *T*, decouples potential differences in growth rates from differences in the quantity of interest, that is, the rates of death. In other words, the decrease in relative abundance of a slow-growing strain will remain constant and will thus not affect the estimation of a change in relative viability (slope), provided that such relative abundance is measured in each outgrowth iteration at the same time after inoculation onto fresh medium.

Let *N_x_* and *N_wt_* be the sizes of two populations, a mutant (*x*) and a wild-type reference (*wt*), growing exponentially in co-culture at rates *g_x_* and *g_wt_*:
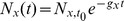


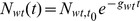
Therefore, the relative number of viable cells from an outgrowth curve inoculated at age *T* (measured in days) in stationary phase and measured at a fixed time point after inoculation (t*, measured in hours), is given by:
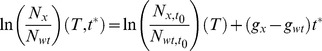
(1)What we get is our measure of interest—the relative number of viable cells at age *T*—plus a constant term that depends on a potential difference in growth rates and which can be ignored. This convenient decoupling would not be possible if the measurement was taken, for instance, at a fixed optical density of the culture. We note that, experimentally, population sizes were large (>10^6^) and all data was collected during exponential growth (see above).

To model aging populations, we consider the simplest model in which mutant (*x*) and reference (*wt*) populations in co-culture die at constant exponential rates [79], *r_x_* and *r_wt_*, resulting in population sizes *N_x_* and *N_wt_* that decay exponentially as a function of age, *T*:
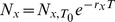


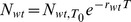
To measure the relative number of viable cells of each population, we take the logarithm of the population ratios as a function of age and obtain the equation for a line, with slope equal to the difference in the death rates of the two populations:
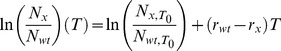
(2)

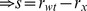
where *s* is the apparent survival coefficient and 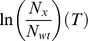 is the quantity that is obtained experimentally by estimating the number of viable cells at different ages of the culture (Equation 1). Since the death rate of the mutant, *r_x_*, is positive, the death rate of the reference strain, *r_wt_*, sets an upper bound for the value of *s* at *s*≤*r_wt_*. We note that, experimentally, only a small number (16 out of 3,878) of CLS measurements exceeded this theoretical upper bound, presumably due to the higher error in the estimate of extreme positive *s* values (λ = NaN; Table S1).

Where needed, we estimated an absolute apparent lifespan value for the mutant from its relative *s* value by combining Equation 2 with the relationship between the death rate and the half life of a population, 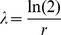, and obtaining an estimate of the mutant's half life:
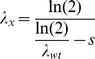
where λ*_wt_* is the measured half life of the RFP-labeled WT strain.

### Genome-wide screen and determination of lifespan factors

A total of 4,340 mutant strains from the yeast deletion collection were successfully tagged; 4,050 reached OD_600_ and RFP signal above an arbitrary threshold. This collection was rearranged in 47 96-well plates which included 414 WT-RFP strains as controls and 48 additional wells with fresh medium or individual cultures of each WT-RFP or CFP-reference to account for background signal. The RFP-labeled deletion collection was screened against a WT CFP-reference strain with a half-life of 21.7 days. After data acquisition and analysis, a relative survival coefficient (corrected *s*) was successfully calculated for 3,878 strains (data in Table S1).

Significantly short- and long-lived strains were scored by assigning a Z-score (*Z*) to each knockout obtained from its *L* value and the standard deviation of 414 *L* measurements for the WT (normally distributed, *p* = 0.48, *x^2^* test). Two-tailed *p*-values were obtained from *Z* to estimate the false discovery rate (FDR) for multiple hypothesis testing; significant phenotypes were assigned using a *q*<0.05 cutoff.

### Functional enrichment analysis

The median relative lifespan (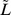) of genes from each Gene Ontology (GO) term was compared to the median relative lifespan of the entire data set (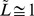). GO terms with an 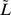 value which was significantly different from that of the genome are shown in Figure 2B (*p*<0.05, Wilcoxon rank sum test). GO annotations were downloaded from the Yeast Genome Database (April 2013); only those GO terms with at least five and no more than 60 genes and genes with a successful *L* measurement and relative growth at *G*>0.9 were considered for analysis.

### Double knockout screen, definition of epistasis, and hierarchical clustering

The final collection of 1,845 double-gene knockouts (41 query strains X 45 array strains) included up to six single-knockout replicates per gene of interest and two marker-swap replicates per double knockout (see correlation in Figure S8). Such independently-measured replicate values of *L*±σ were combined to obtain an error-weighted average relative lifespan, 
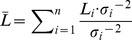
, with standard error 
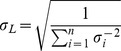
.

For every double deletion of genes *X* and *Y*, epistasis was defined from a multiplicative neutral expectation as: 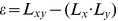, where *L_x_*, *L_y_*, and *L_xy_* are the relative lifespan of the Δ*x* and Δ*y* single knockouts and the Δ*xy* double knockout, respectively. Epistasis error was estimated as 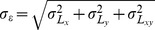. Negative and positive interactions were defined at the 95% confidence interval.

The use of an additive neutral expectation results in a bias to positive epistasis (Figure S10), as has been suggested for epistasis derived from cell growth based phenotypes [59]. We therefore use the conservative multiplicative model in all our epistasis analyses.

Genes sharing similar epistatic-interaction patterns were clustered hierarchically (complete linkage) based on the correlation of raw ε values (Spearman's rank correlation was used to account for strong outliers at both sides of the distribution of ε). Significant interactions and hierarchical clusters were used to generate an epistasis network, which was visualized with Cytoscape 2.8 [80].

### Standard chronological lifespan assay

For validation experiments using the standard CLS method [49], overnight cultures in non-buffered SC (starting from three isolated colonies) were diluted to 0.1 OD units into 25 mL fresh non-buffered SC medium and incubated at 30°C with shaking at 220 rpm in 125 mL flasks covered with aluminum foil. Viability was measured by plating aging cells onto YPD-agar plates and monitoring colony-forming units starting from day 4 after inoculation, which was considered to be the initial survival (100%).

### Fluorescence microscopy

Yeast cells expressing the *CUP1pr-GFP-ATG8* construct were grown to mid-log phase (*OD_600_*≅1) in SC medium. Expression of the fusion protein was induced with 10 mM CuSO_4_ for 2 h, followed by FM4-64 labeling at a final concentration of 10 µg/ml for 20 min. Labeled cells were incubated 90 min in YPD medium to allow for dye uptake and then starved for 7 hours in SD-N medium to induce autophagy. Images were taken using a fluorescence microscope (Leica DM600 B) with a digital camera (Leica DFC420 C).

### Western blotting

The autophagic degradation of Pgk1 was monitored by immunoblotting of the Pgk1-GFP protein fusion as described [54]. Cells were grown overnight at 30°C to stationary phase (*OD_600_*>4) in SC medium. 20 *OD_600_* units of cells were harvested and starved for nitrogen in SD-N medium. At each time point after starvation, 2 *OD_600_* units of cells were lysed with 1.85M NaOH, 7.5% β-mercaptoethanol). Protein extracts were separated on SDS-polyacrylamide gels, transferred to a PVDF membrane, and probed with anti-GFP (Roche Applied Science 11814460001) and secondary antibody (Goat Anti-Mouse IgG coupled to HRP, 31430 Thermo Scientific); reactive band were detected by chemiluminescence (GE Healthcare Life Sciences, RPN2209).

## Supporting Information

Figure S1 Competition-based assays provide replicable CLS data. (**A**) Scatter plot comparing relative lifespan data (*L*) from two independent measurements of 720 deletion strains; *r* is the Pearson's linear correlation coefficient. (**B**) Scatter plot comparing measurements obtained from two biological-replicates. WT and 235 deletion strains (different from those shown in panel *a*) were independently tagged with RFP or CFP and the mutant's relative lifespan was measured in dye-swap experiments.
(PDF)

Figure S2 Competition provides increased quantitative resolution for CLS measurements. The absolute half-life of WT and six gene-deletion strains was measured in replicate (*n* = 24) by monitoring the outgrowth of either individual (gray box plots) or of competing populations (this study, yellow box plots). Outgrowth of individual cultures was monitored by the method of Murakami, *et al.* (2008, *J. Gerontol.* 63:113) adapted to our automated cell-assay station. For each outgrowth culture at a given age, viability was defined as the *OD_600 nm_* reached at a fixed point in time (10 hrs) and normalized to age zero (five days after inoculation); the half life of each strain was defined from the linear regression of *ln*(*OD_600 nm_*) as a function of age (days). For competition-based assays, outgrowth was monitored by relative fluorescence as described and an absolute half life was obtained from *s* (see Materials and Methods). Starting from five days after inoculation, stationary-phase cultures were monitored for 27 days (individual cultures) or 21 days (competition cultures). The dashed line indicates the median half life of the WT (21.7 days).
(PDF)

Figure S3 Relative survival ranks are insensitive to the choice of reference strain. (**A**) Heat map illustrates the relative survival coefficients, *s*, of 24 RFP-labeled deletion strains aged in competition with 16 CFP-labeled strains that span the *s* scale. In the heat map, query and reference strains are ordered from short-lived (bottom left) to long-lived (top right); extreme negative and positive *s* values are thus concentrated at the top left and bottom right. (**B**) Cluster analysis of the Spearman's correlation coefficients (color bar) of the *s* values obtained for each CFP-labeled reference strain (treatment) against all RFP-labeled strains (observations).
(PDF)

Figure S4 Culture aeration has minor effect on CLS estimates. (**A**) CLS phenotypes at low and full aeration are highly correlated. Thirty-two mutant strains were aged in competition to the WT and outgrowth was monitored by relative fluorescence. Low aeration (horizontal axis) corresponds to 700 µl cultures in deep-well plates, as described (see Materials and Methods), while high aeration (vertical axis) corresponds to 8 ml cultures aged in test tubes shaken at 250 rpm and outgrown in microtiter plates; *r* is the Pearson's linear correlation coefficient. (**B**) Semi-automated CLS profiling at high aeration confirms CLS phenotypes at low aeration. Plot shows the cumulative distributions of CLS phenotypes at high aeration for mutant strains defined as neutral, short lived, or long lived under the automated low aeration conditions used in this study. Cultures were aged in deep-well plates shaken at 900 rpm on a Titramax vibratory shaker) Median *L* of both short- or long-lived strains was significantly different from that of neutral mutant strains (*p*<0.05, Wilcoxon rank sum test).
(PDF)

Figure S5 Comparison to growth-rate phenotypes allows identification of CLS-specific genes. (**A**) Short-lived mutant strains are more likely to be affected in their growth phenotype. Plot compares the distribution of growth-rate phenotypes of neutral (black dots) and short-lived mutant strains; *p* is the *p*-value of the Wilcoxon rank sum test. (**B**) Venn diagrams show the numbers of mutant strains with CLS or growth-defect phenotypes; overlap indicates mutants affected in both CLS (short or long lived) and growth rate. CLS phenotypes were from this study (Table S1); *G* is the relative growth rate, expressed as the average value of data obtained from Breslow *et al.* (2008, *Nat. Methods* 5(8):711) and Costanzo *et al.* (2010, *Science* 327(5964):425). A mild/strong growth defect (top) was defined as *G*<0.95, while a strong growth defect (bottom) was *G*<0.9.
(PDF)

Figure S6 Overlap of CLS and growth phenotypes increases only to a small extent under non-fermentative growth conditions. Scatter plots comparing relative lifespan (*L*) in aging medium to growth rate (*G*) in (**A**) fermentative (dextrose) or (**B**) non-fermentative (ethanol+glycerol) yeast-extract peptone medium for a random subset of knockout strains (*n* = 257). Red circles indicate strains with growth defects (*G*<0.9). Labels indicate gene mutations with overlapping CLS and growth phenotypes only under non-fermentative conditions (YPEG). (**C**) Pie charts show the fraction of growth-defect phenotypes under fermentative (YPD, left) or non-fermentative (YPEG) conditions for all tested mutant strains with a short- or long-lived CLS phenotype. Strains were growth in 150 µl of YPD or YPEG medium in 96-well plates shaken at 900 rpm. Absolute growth rates (*gr*) were obtained from the linear fit of *ln*(*OD_600 nm_*) as a function of time; growth relative to the WT is *G* = *gr_x_*/*gr_wt_*.
(PDF)

Figure S7 Our experimental approach can be readily adapted to measure lifespan independently of potential regrowth. Cumulative frequencies of relative lifespan measured in water for mutant strains classified as short-lived (cross signs, *n* = 262) or long-lived (plus signs, *n* = 243). Plot shows strains that were confirmed in water as short-lived (magenta circles) and long-lived strains (cyan circles), based on the distribution of 57 WT-control replicates (95% CI). Strains were diluted into 800 µl of buffered SC medium in semi-deep well plates and transferred to water five days after inoculation. Every three days, cultures were washed twice in water by microplate centrifugation. Cultures were monitored for relative survival during 21 days and relative lifespan was calculated as described (see Materials and Methods).
(PDF)

Figure S8Characterization of single and double knockouts for epistasis analysis. (**A**). Plot shows the average relative lifespan (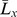) of single autophagy mutants in which each mutant's 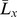 value (black circles) was derived from two to up to six independent single mutant replicates; error bars are the standard error. Single-knockout strains in the horizontal axis are ranked from largest to smallest 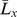. (**B**) Scatter plot compares *L* data from two marker-swap replicates available for each double-gene knockout; *r* is the Pearson's linear correlation coefficient.(PDF)

Figure S9 Most single mutants used for epistasis analysis are unaffected in their exponential-growth rates. (**A**) Strains were grown in 150 µl of YPD medium in 96-well plates shaken at 900 rpm. Absolute growth rates (*gr*) were obtained from the linear fit of *ln*(*OD_600 nm_*) as a function of time during exponential phase. (**B**) Histogram shows the growth rates for all single knockouts relative to the WT, calculated as *G* = *gr_x_*/*gr_WT_*.
(PDF)

Figure S10Comparing the spectrum of epistasis derived from different neutral expectations. Epistasis was defined from (**A**) a multiplicative neutral expectation, 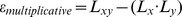, or (**B**) an additive neutral expectation, 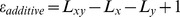. In each case, the spectrum of epistasis is shown for interactions among non-core-autophagy (blue histograms) and core-autophagy genes (purple histogram outline, *n* = 75). Pie charts show the fraction of significant negative (ε<0) and positive (ε>0) interaction pairs, for both the non-core (top) and core-autophagy gene sets (bottom).(PDF)

Figure S11 Relative-fluorescence dynamic range. Scatter plots show the observed (lnR/C^t*^) fluorescent signal versus the expected (*lnR/C*) signal (defined dilutions of RFP- and CFP-labeled WT cells). Panels (**A**) and (**B**) show experiments done with different dilution rates of the two populations. Values represent the mean of eight independent experimental replicates and its standard deviation (error bars). Red dotted lines depict conservative upper and lower lnR/C^t*^ thresholds employed to select the per-day lnR/C^t*^ measured values in the genome wide CLS assay. Expected fluorescent signal is equal to defined dilutions of RFP-marked WT strain in the CFP-marker WT strain and the signal detected from these dilutions at a fixed time point post inoculation, t* correspond to the observed fluorescent signal.
(PDF)

Figure S12 Data normalization. Representative scatter plot of raw survival coefficients, *s* data obtained from CLS measurements of single and double-knockout mutants arranged on 44 batches (two batches per 96-well plate), before (**A**) and after (**B**) per plate-mean normalization (corrected *s*). Normalization was made by subtracting the mean value of eight WT-control samples per batch to all raw *s* values in each batch. Blue dots represent WT replicates, light-blue dots are single deletions, and gray dots are double-deletion strains.
(PDF)

Table S1 Genome-wide CLS screen, complete data set. Relative growth rate, *G*, is the average of data from Breslow *et al.* (2008, *Nat. Methods* 5(8):711) and Costanzo *et al.* (2010, *Science* 327(5964):425).
(XLS)

Table S2 Lists of long-lived and short-lived single knockouts. A filter of *G*>0.95 was applied for short-lived strains. No growth filter was used for long-lived strains. *L_water_* = 1 indicates that the longevity phenotype was confirmed in water.
(XLSX)

Table S3 Lifespan-epistasis data for autophagy genes. Interactions indicate positive or negative epistasis at the 95% CI.
(XLS)
