# Supplementary figures and images for: High-Resolution Profiling of Stationary-Phase Survival Reveals Yeast Longevity Factors and Their Genetic Interactions
Source: PLoS Genet. 2014 Feb 27;10(2):e1004168. doi: 10.1371/journal.pgen.1004168 (PMC3937222; doi:10.1371/journal.pgen.1004168)

**a**

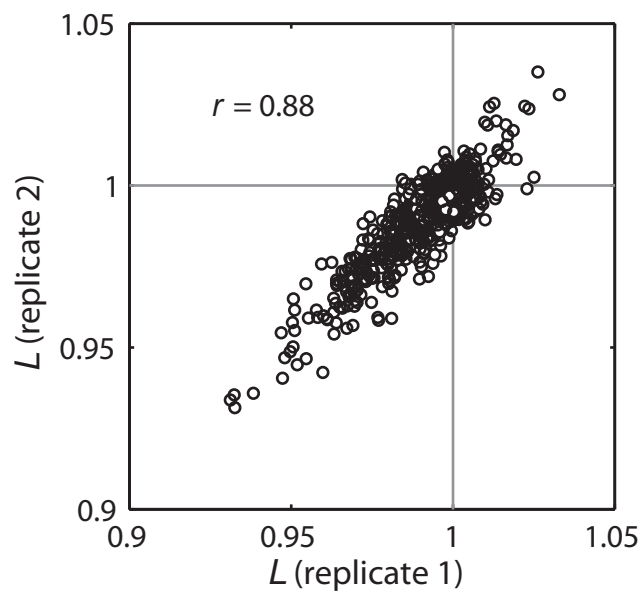

**b**

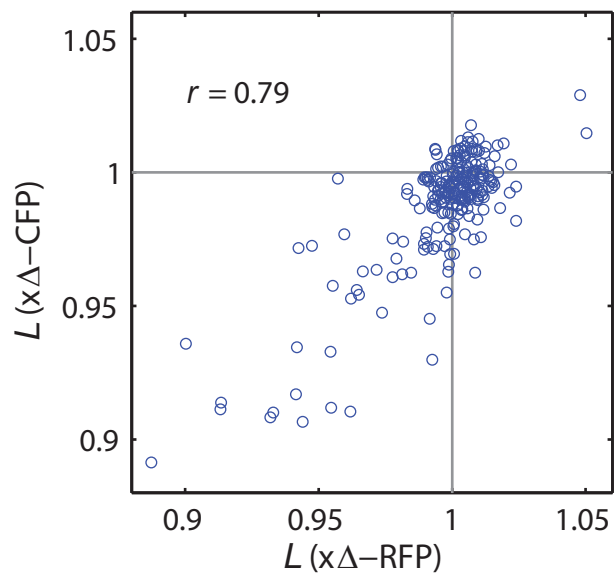

Supplement: Figure S1 — Competition-based assays provide replicable CLS data. (A) Scatter plot comparing relative lifespan data (L) from two independent measurements of 720 deletion strains; r is the Pearson's linear correlation coefficient. (B) Scatter plot comparing measurements obtained from two biological-replicates. WT and 235 deletion strains (different from those shown in panel a) were independently tagged with RFP or CFP and the mutant's relative lifespan was measured in dye-swap experiments. (PDF) [file pgen.1004168.s001.pdf]

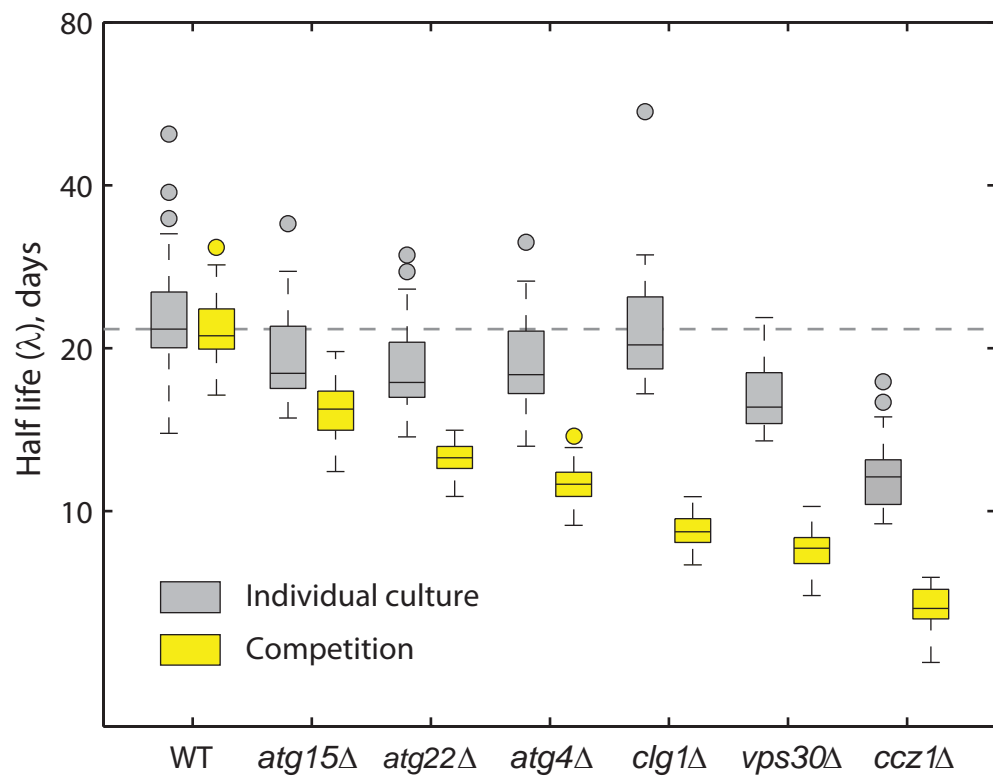

Supplement: Figure S2 — Competition provides increased quantitative resolution for CLS measurements. The absolute half-life of WT and six gene-deletion strains was measured in replicate (n = 24) by monitoring the outgrowth of either individual (gray box plots) or of competing populations (this study, yellow box plots). Outgrowth of individual cultures was monitored by the method of Murakami, et al. (2008, J. Gerontol. 63:113) adapted to our automated cell-assay station. For each outgrowth culture at a given age, viability was defined as the OD600 nm reached at a fixed point in time (10 hrs) and normalized to age zero (five days after inoculation); the half life of each strain was defined from the linear regression of ln(OD600 nm) as a function of age (days). For competition-based assays, outgrowth was monitored by relative fluorescence as described and an absolute half life was obtained from s (see Materials and Methods). Starting from five days after inoculation, stationary-phase cultures were monitored for 27 days (individual cultures) or 21 days (competition cultures). The dashed line indicates the median half life of the WT (21.7 days). (PDF) [file pgen.1004168.s002.pdf]

a

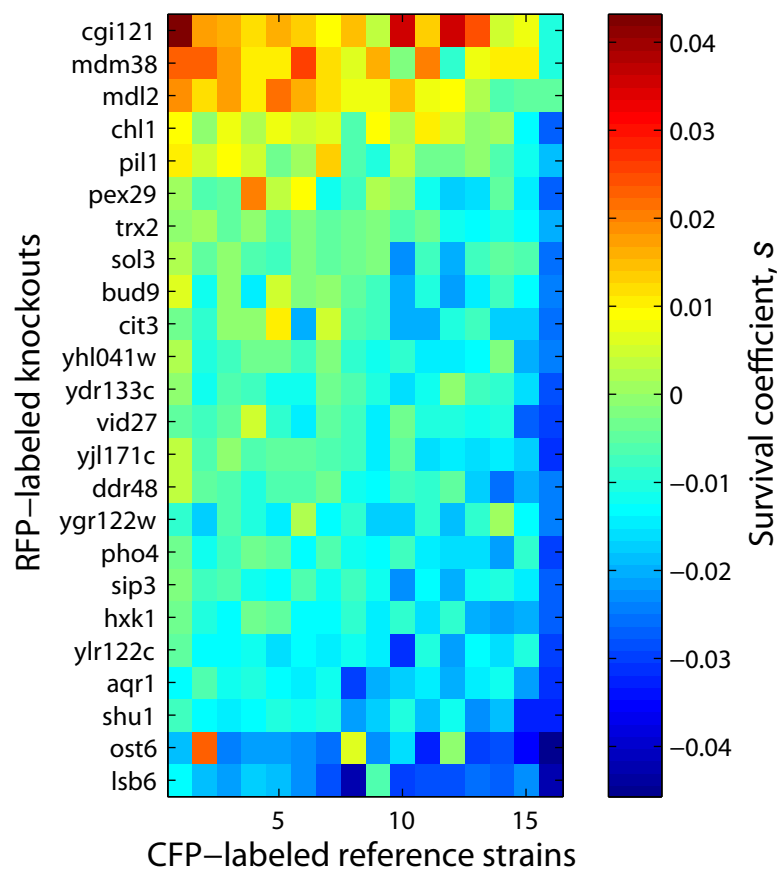

b

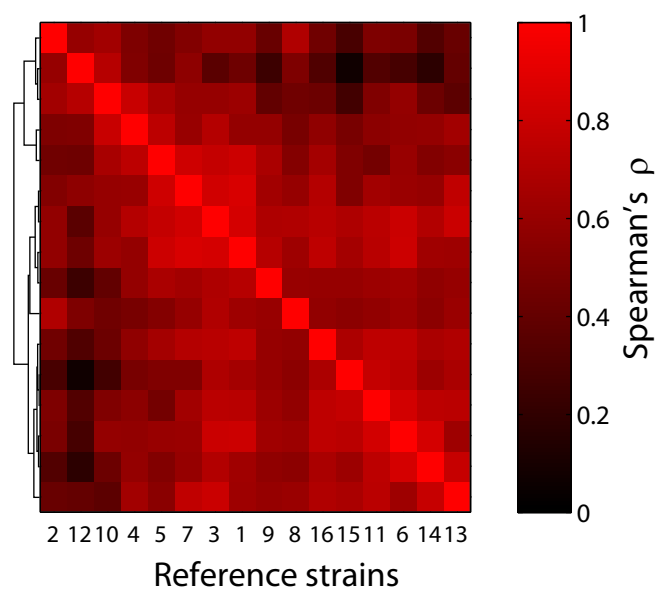

Supplement: Figure S3 — Relative survival ranks are insensitive to the choice of reference strain. (A) Heat map illustrates the relative survival coefficients, s, of 24 RFP-labeled deletion strains aged in competition with 16 CFP-labeled strains that span the s scale. In the heat map, query and reference strains are ordered from short-lived (bottom left) to long-lived (top right); extreme negative and positive s values are thus concentrated at the top left and bottom right. (B) Cluster analysis of the Spearman's correlation coefficients (color bar) of the s values obtained for each CFP-labeled reference strain (treatment) against all RFP-labeled strains (observations). (PDF) [file pgen.1004168.s003.pdf]

**a**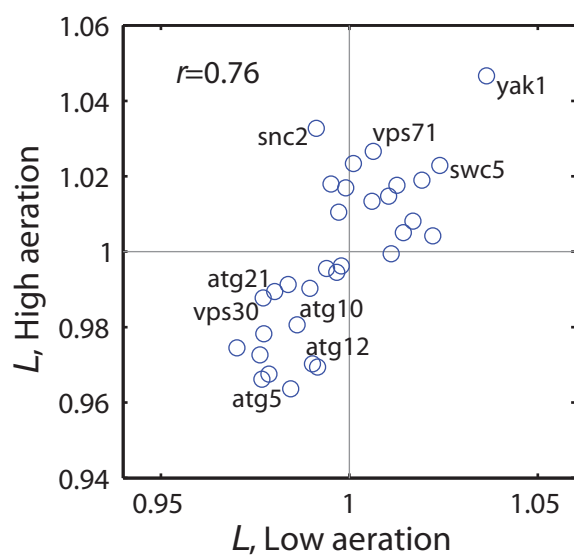**b**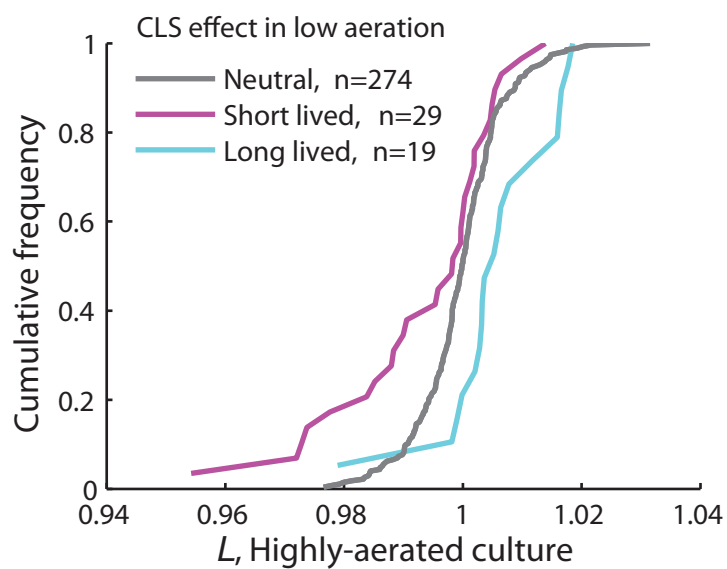

Supplement: Figure S4 — Culture aeration has minor effect on CLS estimates. (A) CLS phenotypes at low and full aeration are highly correlated. Thirty-two mutant strains were aged in competition to the WT and outgrowth was monitored by relative fluorescence. Low aeration (horizontal axis) corresponds to 700 µl cultures in deep-well plates, as described (see Materials and Methods), while high aeration (vertical axis) corresponds to 8 ml cultures aged in test tubes shaken at 250 rpm and outgrown in microtiter plates; r is the Pearson's linear correlation coefficient. (B) Semi-automated CLS profiling at high aeration confirms CLS phenotypes at low aeration. Plot shows the cumulative distributions of CLS phenotypes at high aeration for mutant strains defined as neutral, short lived, or long lived under the automated low aeration conditions used in this study. Cultures were aged in deep-well plates shaken at 900 rpm on a Titramax vibratory shaker) Median L of both short- or long-lived strains was significantly different from that of neutral mutant strains (p<0.05, Wilcoxon rank sum test). (PDF) [file pgen.1004168.s004.pdf]

**a**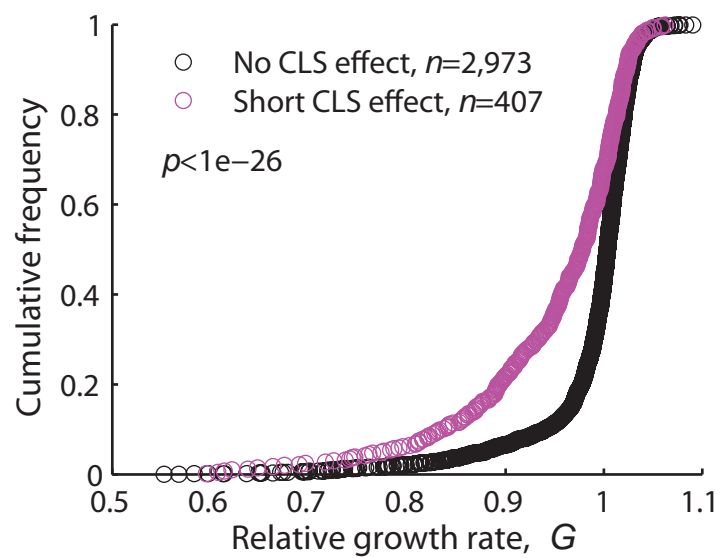**b**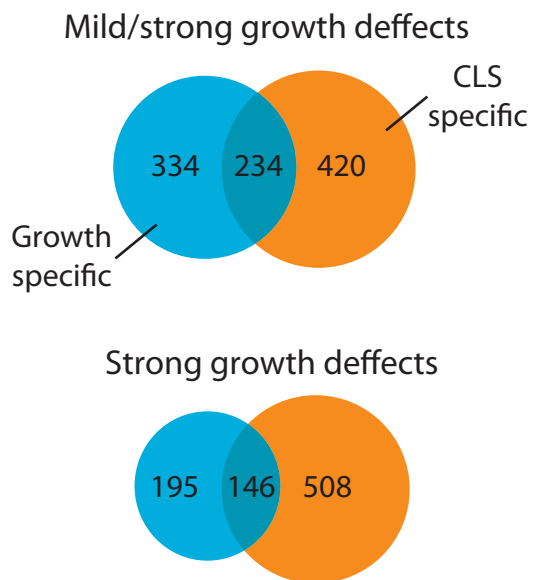

Supplement: Figure S5 — Comparison to growth-rate phenotypes allows identification of CLS-specific genes. (A) Short-lived mutant strains are more likely to be affected in their growth phenotype. Plot compares the distribution of growth-rate phenotypes of neutral (black dots) and short-lived mutant strains; p is the p-value of the Wilcoxon rank sum test. (B) Venn diagrams show the numbers of mutant strains with CLS or growth-defect phenotypes; overlap indicates mutants affected in both CLS (short or long lived) and growth rate. CLS phenotypes were from this study (Table S1); G is the relative growth rate, expressed as the average value of data obtained from Breslow et al. (2008, Nat. Methods 5(8):711) and Costanzo et al. (2010, Science 327(5964):425). A mild/strong growth defect (top) was defined as G<0.95, while a strong growth defect (bottom) was G<0.9. (PDF) [file pgen.1004168.s005.pdf]

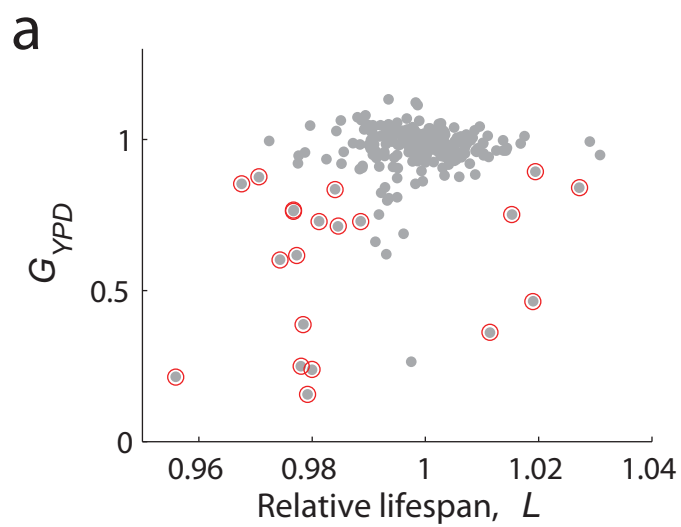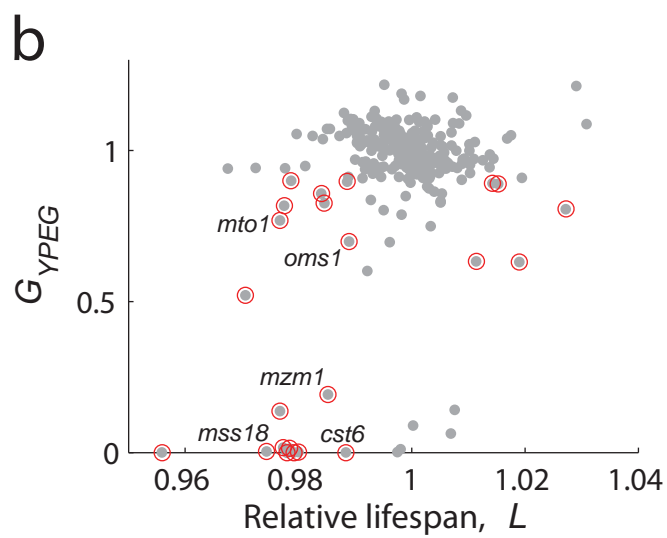

**c**

Gene-knockouts with CLS effect

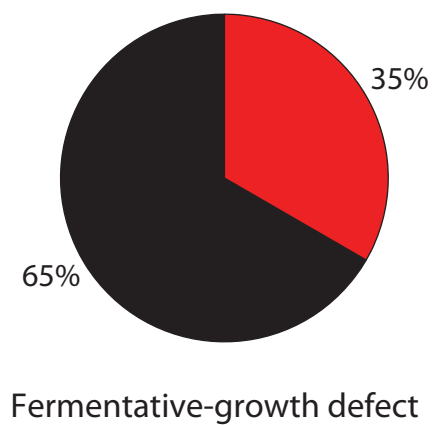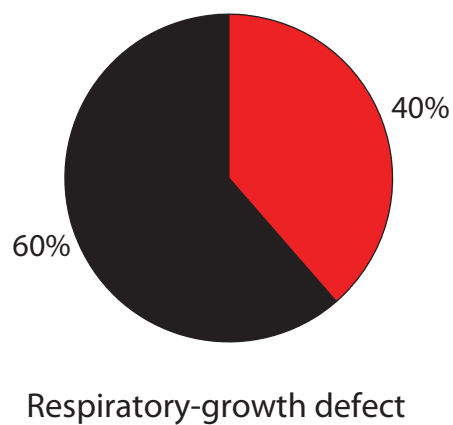

Supplement: Figure S6 — Overlap of CLS and growth phenotypes increases only to a small extent under non-fermentative growth conditions. Scatter plots comparing relative lifespan (L) in aging medium to growth rate (G) in (A) fermentative (dextrose) or (B) non-fermentative (ethanol+glycerol) yeast-extract peptone medium for a random subset of knockout strains (n = 257). Red circles indicate strains with growth defects (G<0.9). Labels indicate gene mutations with overlapping CLS and growth phenotypes only under non-fermentative conditions (YPEG). (C) Pie charts show the fraction of growth-defect phenotypes under fermentative (YPD, left) or non-fermentative (YPEG) conditions for all tested mutant strains with a short- or long-lived CLS phenotype. Strains were growth in 150 µl of YPD or YPEG medium in 96-well plates shaken at 900 rpm. Absolute growth rates (gr) were obtained from the linear fit of ln(OD600 nm) as a function of time; growth relative to the WT is G = grx/grwt. (PDF) [file pgen.1004168.s006.pdf]

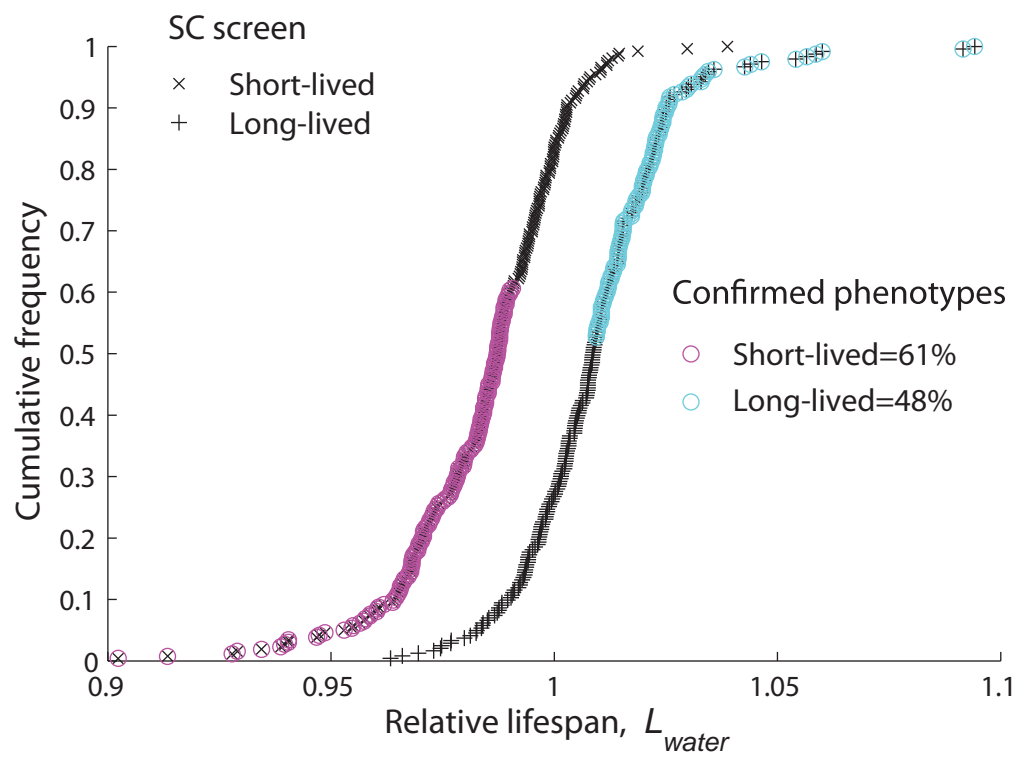

Supplement: Figure S7 — Our experimental approach can be readily adapted to measure lifespan independently of potential regrowth. Cumulative frequencies of relative lifespan measured in water for mutant strains classified as short-lived (cross signs, n = 262) or long-lived (plus signs, n = 243). Plot shows strains that were confirmed in water as short-lived (magenta circles) and long-lived strains (cyan circles), based on the distribution of 57 WT-control replicates (95% CI). Strains were diluted into 800 µl of buffered SC medium in semi-deep well plates and transferred to water five days after inoculation. Every three days, cultures were washed twice in water by microplate centrifugation. Cultures were monitored for relative survival during 21 days and relative lifespan was calculated as described (see Materials and Methods). (PDF) [file pgen.1004168.s007.pdf]

**a**

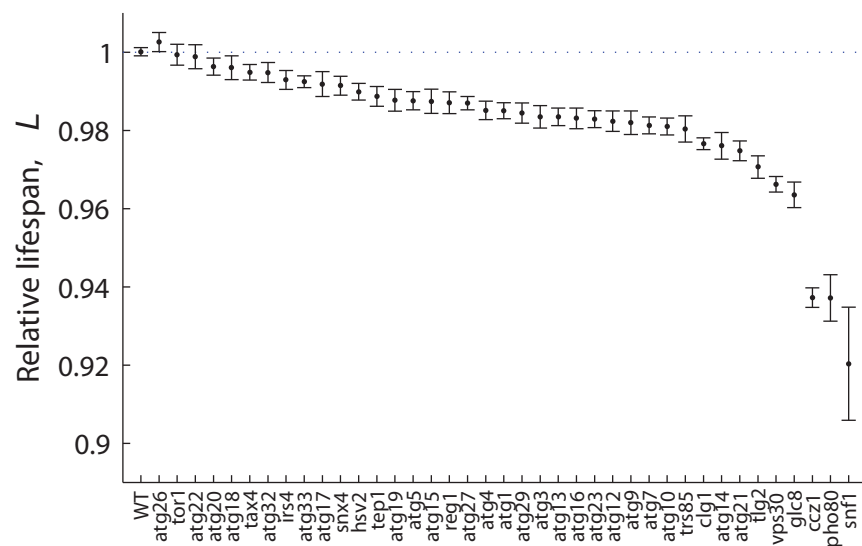

**b**

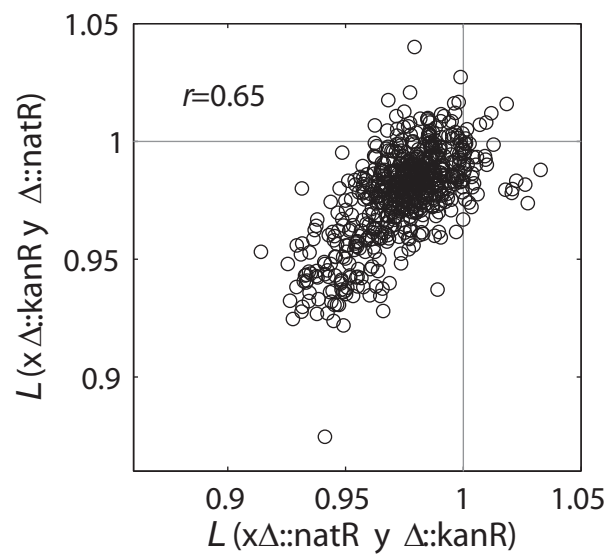

Supplement: Figure S8 — Characterization of single and double knockouts for epistasis analysis. (A). Plot shows the average relative lifespan () of single autophagy mutants in which each mutant's value (black circles) was derived from two to up to six independent single mutant replicates; error bars are the standard error. Single-knockout strains in the horizontal axis are ranked from largest to smallest . (B) Scatter plot compares L data from two marker-swap replicates available for each double-gene knockout; r is the Pearson's linear correlation coefficient. (PDF) [file pgen.1004168.s008.pdf]

a

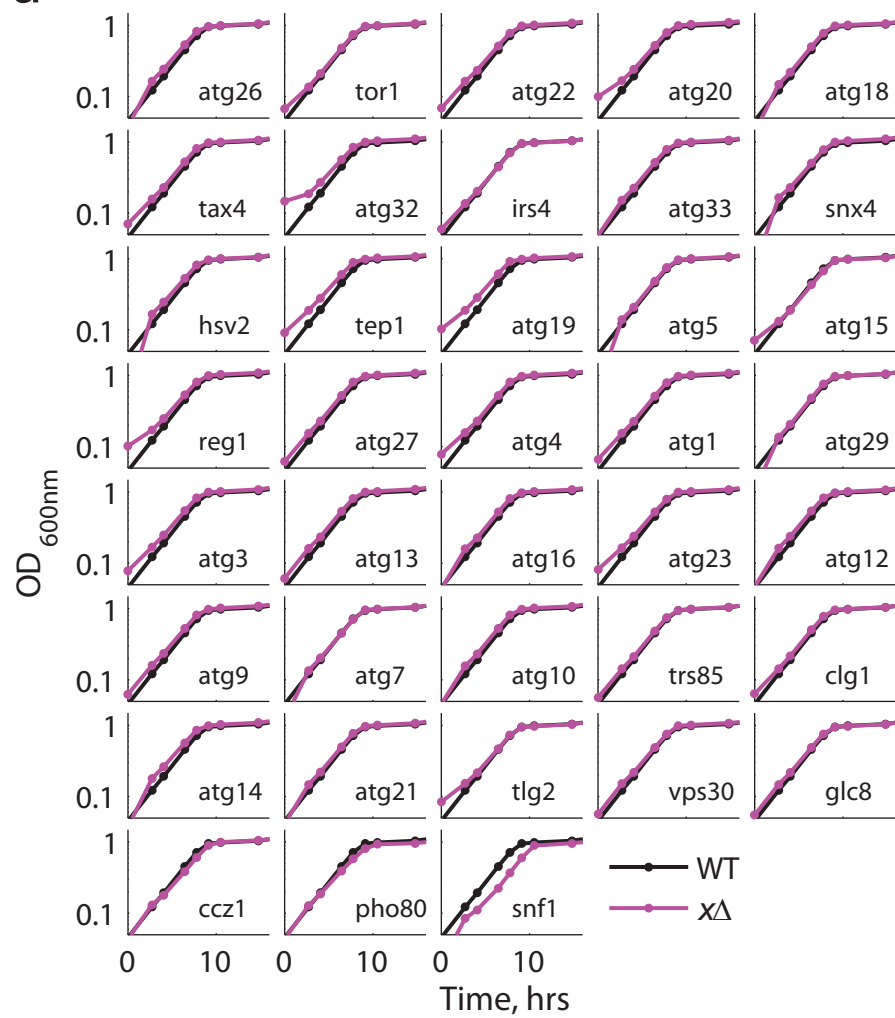

b

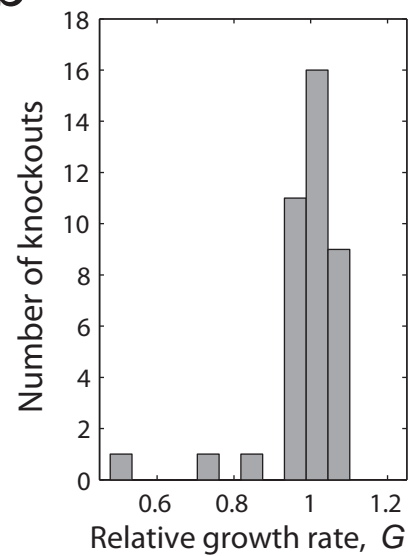

Supplement: Figure S9 — Most single mutants used for epistasis analysis are unaffected in their exponential-growth rates. (A) Strains were grown in 150 µl of YPD medium in 96-well plates shaken at 900 rpm. Absolute growth rates (gr) were obtained from the linear fit of ln(OD600 nm) as a function of time during exponential phase. (B) Histogram shows the growth rates for all single knockouts relative to the WT, calculated as G = grx/grWT. (PDF) [file pgen.1004168.s009.pdf]

**a**

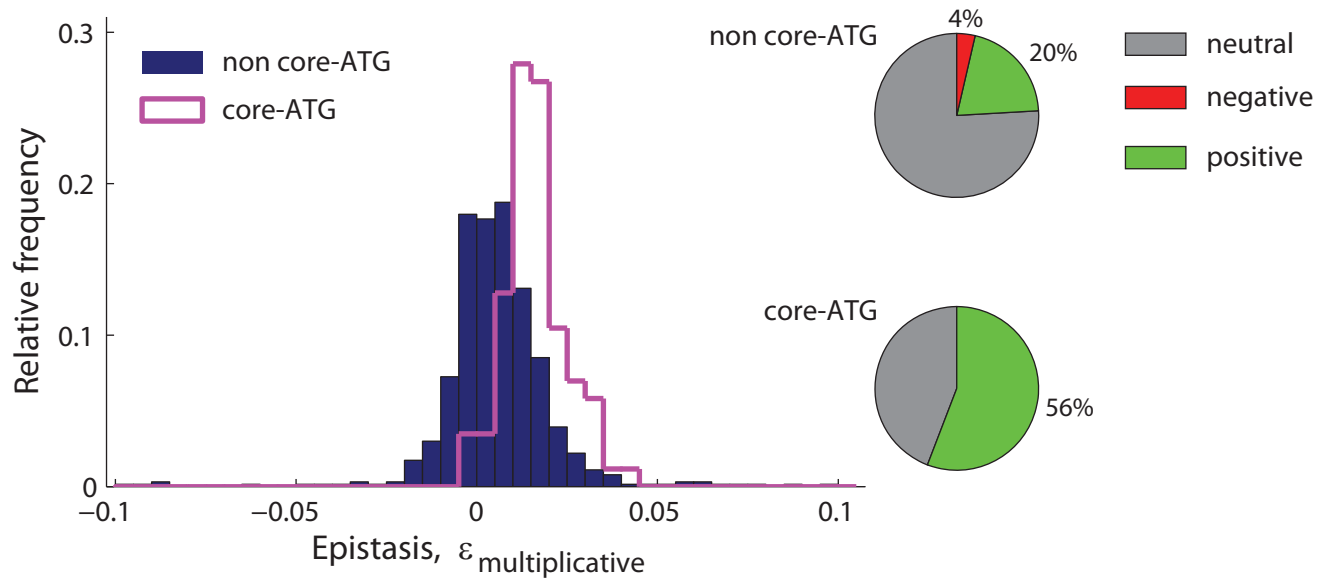

**b**

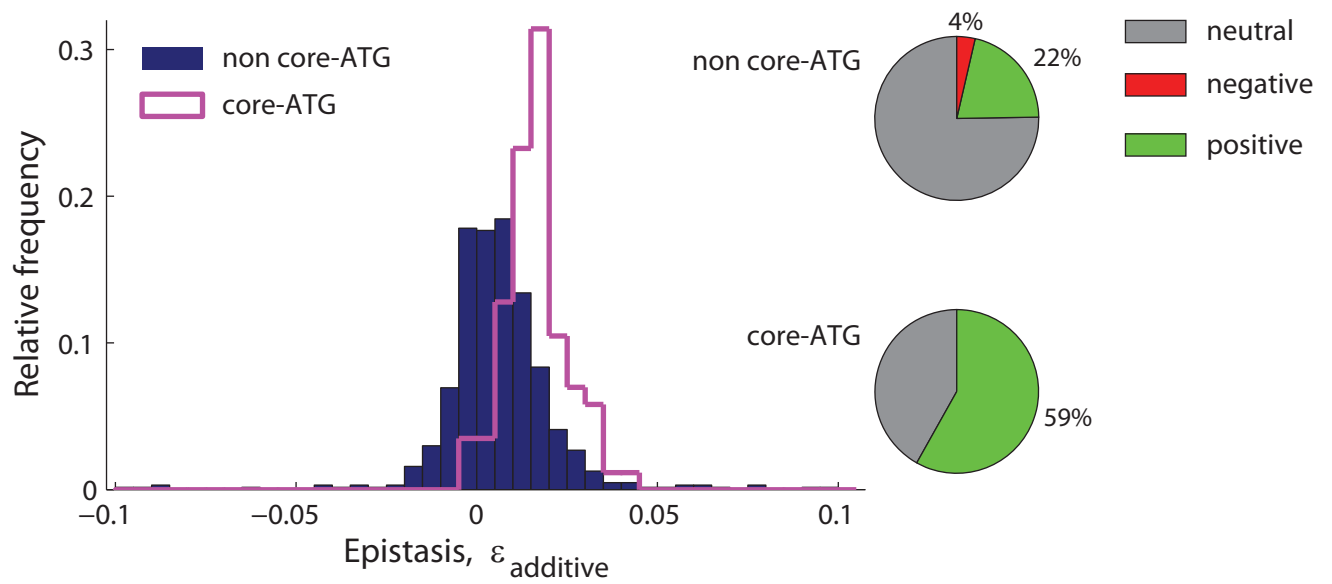

Supplement: Figure S10 — Comparing the spectrum of epistasis derived from different neutral expectations. Epistasis was defined from (A) a multiplicative neutral expectation, , or (B) an additive neutral expectation, . In each case, the spectrum of epistasis is shown for interactions among non-core-autophagy (blue histograms) and core-autophagy genes (purple histogram outline, n = 75). Pie charts show the fraction of significant negative (ε<0) and positive (ε>0) interaction pairs, for both the non-core (top) and core-autophagy gene sets (bottom). (PDF) [file pgen.1004168.s010.pdf]

a

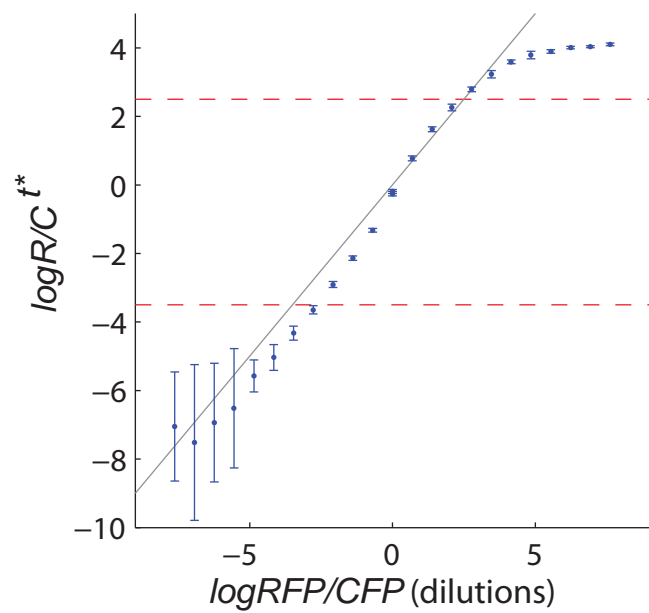

b

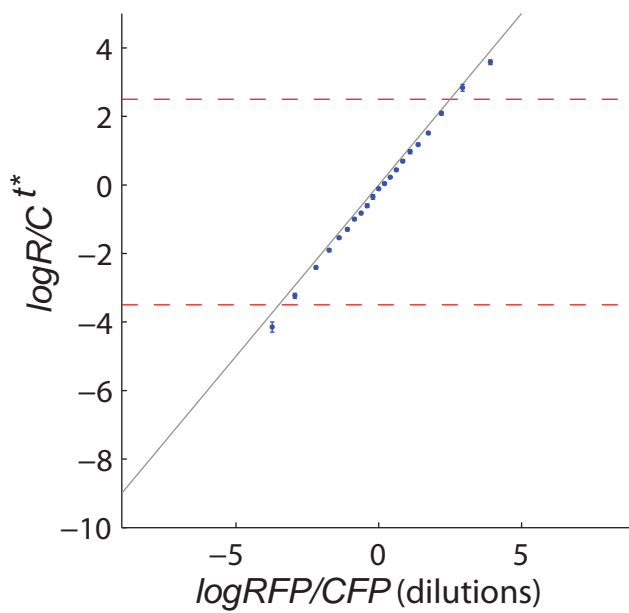

Supplement: Figure S11 — Relative-fluorescence dynamic range. Scatter plots show the observed (lnR/Ct*) fluorescent signal versus the expected (lnR/C) signal (defined dilutions of RFP- and CFP-labeled WT cells). Panels (A) and (B) show experiments done with different dilution rates of the two populations. Values represent the mean of eight independent experimental replicates and its standard deviation (error bars). Red dotted lines depict conservative upper and lower lnR/Ct* thresholds employed to select the per-day lnR/Ct* measured values in the genome wide CLS assay. Expected fluorescent signal is equal to defined dilutions of RFP-marked WT strain in the CFP-marker WT strain and the signal detected from these dilutions at a fixed time point post inoculation, t* correspond to the observed fluorescent signal. (PDF) [file pgen.1004168.s011.pdf]

a

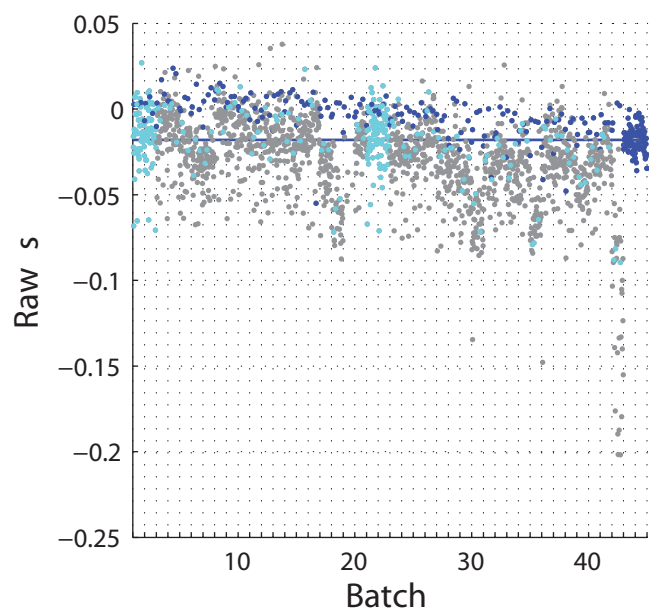

b

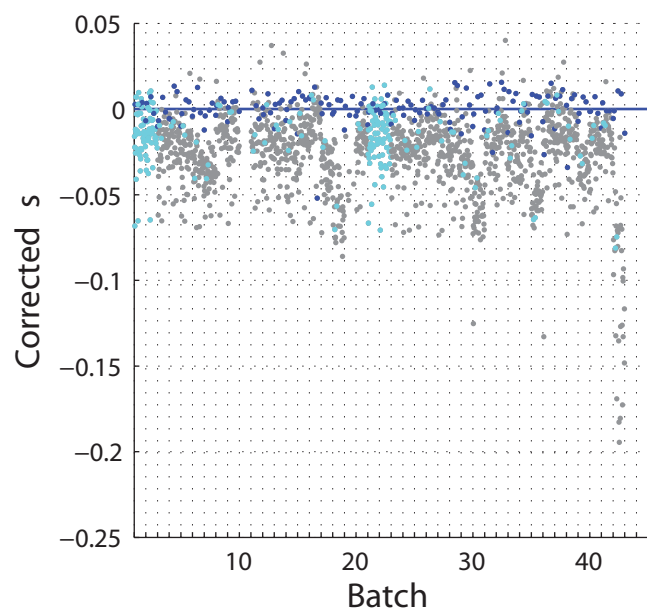

Supplement: Figure S12 — Data normalization. Representative scatter plot of raw survival coefficients, s data obtained from CLS measurements of single and double-knockout mutants arranged on 44 batches (two batches per 96-well plate), before (A) and after (B) per plate-mean normalization (corrected s). Normalization was made by subtracting the mean value of eight WT-control samples per batch to all raw s values in each batch. Blue dots represent WT replicates, light-blue dots are single deletions, and gray dots are double-deletion strains. (PDF) [file pgen.1004168.s012.pdf]
